# Assessing the Effectiveness of Eco-Friendly Management Approaches for Controlling Wheat Yellow Rust and Their Impact on Antioxidant Enzymes

**DOI:** 10.3390/plants12162954

**Published:** 2023-08-15

**Authors:** Waleed Gamal Eldein Zakaria, Mahmoud Mohamed Atia, Ahmed Zaki Ali, Entsar E. A. Abbas, Bilkess M. A. Salim, Samy A. Marey, Ashraf Atef Hatamleh, Ahmed Saeed Mohammed Elnahal

**Affiliations:** 1 Department of Plant Pathology, Faculty of Agriculture, Zagazig University, Zagazig 44511, Egypt; waleed.g.m84@gmail.com (W.G.E.Z.); usamaatia2@zu.edu.eg (M.M.A.); entsarabbas@zu.edu.eg (E.E.A.A.); 2 State Key Laboratory of Crop Stress Biology for Arid Areas, College of Plant Protection, Northwest A&F University, Xianyang 712100, China; 3 Plant Production Department, Faculty of Agriculture, Sabha University, Sabha P.O. Box 18758, Libya; malikaa62@gmail.com; 4 King Saud University, Riyadh 11451, Saudi Arabia; samarey@ksu.edu.sa; 5 Department of Botany and Microbiology, College of Science, King Saud University, Riyadh 11451, Saudi Arabia; ahatamleh@ksu.edu.sa

**Keywords:** wheat, stripe rust, *Puccinia striiformis*, integrated management, *B. subtilis*, *P. putida*, chemical inducers, varietal reactions, fungicides, antioxidant enzymes

## Abstract

Wheat stripe rust, caused by *Puccinia striiformis* f. sp. *tritici* (*Pst*), is a destructive disease that causes significant yield losses in wheat production worldwide, including in Egypt. The use of biocontrol agents is among the best eco-friendly management strategies to control this disease, as they are more sustainable and environmentally friendly than traditional chemical control methods. In a comparative analysis, antioxidant enzyme activity and various management approaches were compared with two bacterial biocontrol agents, *Bacillus subtilis* and *Pseudomonas putida*. This study showed the remarkable efficacy of endophytic bacteria, *B. subtilis* and *P. putida*, in mitigating wheat stripe rust infection across three wheat varieties, namely Misr1, Gimmeiza11, and Sids12. *B. subtilis* exhibited superior performance compared to *P. putida*, resulting in infection types of 1 and 2.66, respectively, following inoculation. The highest reduction rate was observed with Tilit fungicide (500 ppm), followed by B. subtilis and Salicylic acid (1000 ppm), respectively. Variations in wheat varieties’ response to *Pst* infection were observed, with Misr1 exhibiting the lowest infection and Sids12 showing high susceptibility. Among the tested inducers, Salicylic acid demonstrated the greatest reduction in disease infection, followed by Indole acetic acid, while Oxalic acid exhibited the lowest decrease. Additionally, the study evaluated the activities of five antioxidant enzymes, including Catalase, Ascorbate peroxidase (APX), glutathione reductase (GR), Superoxide dismutase (SOD), and peroxidase (POX), in the wheat-stripe rust interaction under different integrated management approaches. The wheat variety Misr1 treated with Tilit (500 ppm), B. subtilis, Salicylic acid, Montoro (500 ppm), and *P. putida* exhibited the highest increase in all enzymatic activities. These findings provide valuable insights into the effectiveness of *B. subtilis* and *P. putida* as biocontrol agents for wheat stripe rust control in Egypt, emphasizing their potential role in sustainable, integrated, and environmentally friendly management practices.

## 1. Introduction

Wheat yellow rust (WYR) or stripe rust (SR), caused by Puccinia striiformis f. sp. tritici (*Pst*), presents a substantial obstacle to wheat production, resulting in severe damage to wheat crops globally [1,2,3], particularly in regions with wet and cool weather [3]. The *Pst* has the ability to migrate vast distances and mutate itself to cope with diverse climatic conditions [4]. Consequently, it is critical to spot this disease early on. In Egypt, where wheat is a crucial staple crop, the impact of this disease can be particularly severe, leading to significant economic losses and compromising food security [5].

The disease adversely impacted grain yield output at most Egyptian wheat varieties. The disease has been reported in Egypt since 1995, producing grain yield losses ranging from 14% to 26% in the Nile Delta area, with a national loss of 10% [6]. Recently, several epidemics have occurred due to the development and spread of novel pathogenic strains of the pathogen, which can surpass the resistance genes of most wheat cultivars [7]. For example, a set of commercial cultivars in Egypt, i.e., Sakha61, Giza171, and Misr2, became susceptible under field conditions after being resistant to the previously identified races [8].

Traditional methods of controlling WYR disease have relied on the use of chemical pesticides, which can have adverse environmental impacts and contribute to the development of pesticide resistance [9]. Fungicides for WYR control are extensively employed in many regions of the world for high production, and their efficiency is dependent on the crop stage and disease severity [10,11]. Sadly, improper usage of chemical pesticides causes deadly environmental contamination due to their lasting effects. Ecofriendly management of WYR involves implementing integrated disease management strategies, including resistant cultivars, biological control, cultural methods, induced resistance, and fungicide application [12]. 

Developing resistant varieties is the best way to prevent this disease; however, the disease can get beyond the wheat varieties’ resistance. The pathogen’s high pathogenicity variation allows its population to grow fast and spread under favorable climatic circumstances, culminating in catastrophic pandemics within cropping seasons [13]. Yet, wheat breeding techniques are not sufficient for coping with the newly developed *Pst* strains and developing wheat varieties with high yields and resistance to WYR disease [11]. Nevertheless, using resistant types alone may not be adequate to entirely prevent yellow rust; other management measures may be required [12].

Using bioagents or endophytic bacteria can be a promising approach for controlling WYR [11]. These naturally occurring microorganisms can help protect the plant from fungal diseases by competing with the pathogen for nutrients or producing antifungal compounds [14]. This approach can be particularly useful for reducing the reliance on chemical pesticides and promoting more sustainable agriculture. Using bioagents or endophytic bacteria as eco-friendly management approaches for controlling WYR disease can have several benefits beyond the reduction of chemical pesticide usage [15]. For example, these approaches can also promote soil health and biodiversity by maintaining a healthy microbial community in the soil [16]. Additionally, using bioagents can be a cost-effective and sustainable approach to controlling yellow rust, especially for small-scale farmers who may not have access to expensive chemical pesticides [17].

Chemical inducers like salicylic and indole acetic acid can also be used to enhance the plant’s resistance to yellow rust [18]. These compounds can activate the plant’s natural defense mechanisms, making it more resistant to the fungal pathogen. While the use of chemical inducers may not eliminate the need for fungicides, it can reduce the amount of pesticides needed and promote more sustainable and environmentally friendly agriculture [19]. The use of chemical inducers like salicylic, chitosan, abscisic acid, indole acetic acid, and indole 3-butyric acid can also have additional benefits beyond controlling yellow rust [18,20]. These compounds can stimulate plant growth and improve plant resilience to stresses, as well as activation of the host’s defense mechanism that occurs through the upregulation of PR protein genes, which serve as crucial players in inducing resistance when triggered by chemical inducers [18,21]. 

The objective of this study is to assess the effectiveness of eco-friendly management approaches for controlling WYR in Egypt and their impact on antioxidant enzymes. We aim to identify the most effective and sustainable strategies for controlling yellow rust in wheat. Our study will also provide insights into the mechanisms underlying the effectiveness of these approaches, which can help inform the development of new eco-friendly management strategies for controlling yellow rust. Ultimately, our goal is to promote more sustainable and environmentally friendly agriculture in Egypt and beyond while also contributing to food security and the long-term sustainability of wheat production.

## 2. Results

### 2.1. Wheat Stripe Rust Infection Type upon Application of Two Tested Bacterial Bioagents

**Figure 1:**
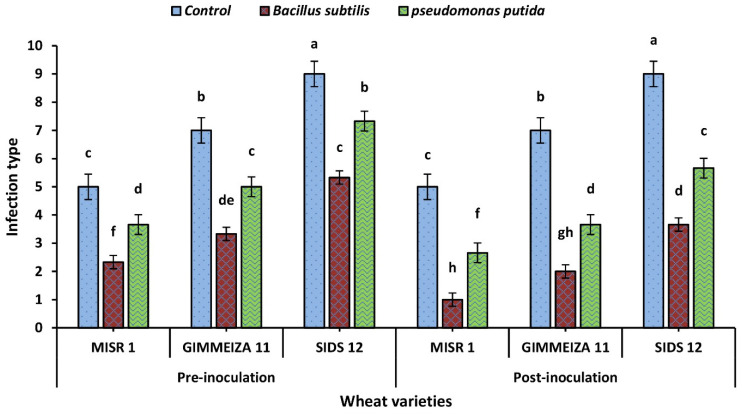
Effect of two endophytic bacteria (bioagents), including *B. subtilis* and *P. putida*, on stripe rust infection in three wheat varieties (Misr1, Gimmeiza11, and Sids12): Pre- and post-inoculation with *Pst* pathogen. The assessment of infection type was performed 20 days after *Pst* inoculation. The infection type was recorded based on the scale described by Chen [22]. The letters assigned (e.g., ‘a’, ‘b’, ‘c’) correspond to the results of Duncan’s multiple range test conducted at a significance level of *p* < 0.05.

The three wheat varieties were assessed for SR infection type before and after inoculation with two endophytic bacteria, *B. subtilis* and *P. putida*, as indicated in Figure 1. The results indicated that the stripe rust infection type was reduced after inoculation with the endophytic bacteria for these varieties. For the Misr1 wheat variety, the stripe rust infection type was 2.33 before inoculation with *B. subtilis* and was reduced to 1 after inoculation. With *P. putida*, the infection type was 3.66 before inoculation and 2.66 after inoculation. The Gimmeiza11 wheat variety had a stripe rust infection type of 3.33 before inoculation with *B. subtilis*, which was reduced to 2 after inoculation. When inoculated with *P. putida*, the infection type was 5 before inoculation and 3.66 after inoculation. Lastly, the Sids12 wheat variety had the highest infection type before inoculation, which was 5.33 with *B. subtilis* and 7.33 with *P. putida*. After inoculation, the infection type was reduced to 3.66 and 5.66, respectively. 

**Figure 2:**
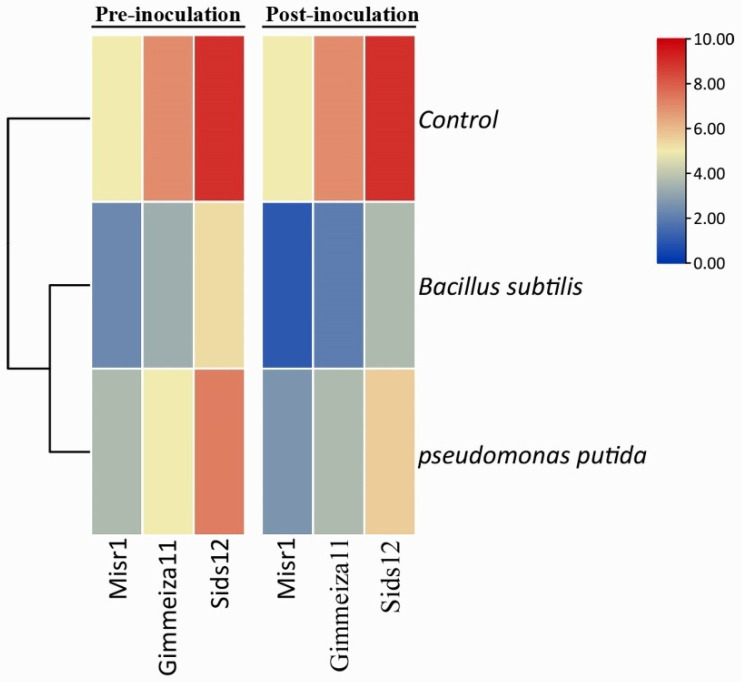
Heat map shows the effect of biocontrol agents against stripe rust infection in three different wheat varieties, including Misr1, Gimmeiza11, and sids12. The heat map is divided into two sections, pre-inoculation and post-inoculation, with the pathogen *Pst*.

Additionally, the impact of both bioagents against stripe rust infection in the three tested wheat varieties is shown in a heat map in Figure 2. The heat map is divided into two portions, pre-inoculation and post-inoculation, with the pathogen *Pst*. The heat map is color-coded, with red denotes a high degree of infection, whereas blue denotes a low level of infection. The heat map of the control group exhibited a substantially high level of infection in all tested varieties. Notably, Sids12 exhibited the highest infection, represented by a darkened red color, followed by Gimmeiza11, whereas Misr1 displayed a lower infection level. Notably, the level of infection decreased in all three varieties when treated with both bacterial bio-agents. Overall, the heat map demonstrates that *B. subtilis* proves to be a more effective bioagent than *P. putida* in reducing stripe rust infection in wheat. Additionally, the heat map highlights that the two bioagents are most effective when applied after the wheat plants are inoculated with *Pst*.

### 2.2. Wheat Varietal Reaction in Response to Stripe Rust Infection

**Figure 3:**
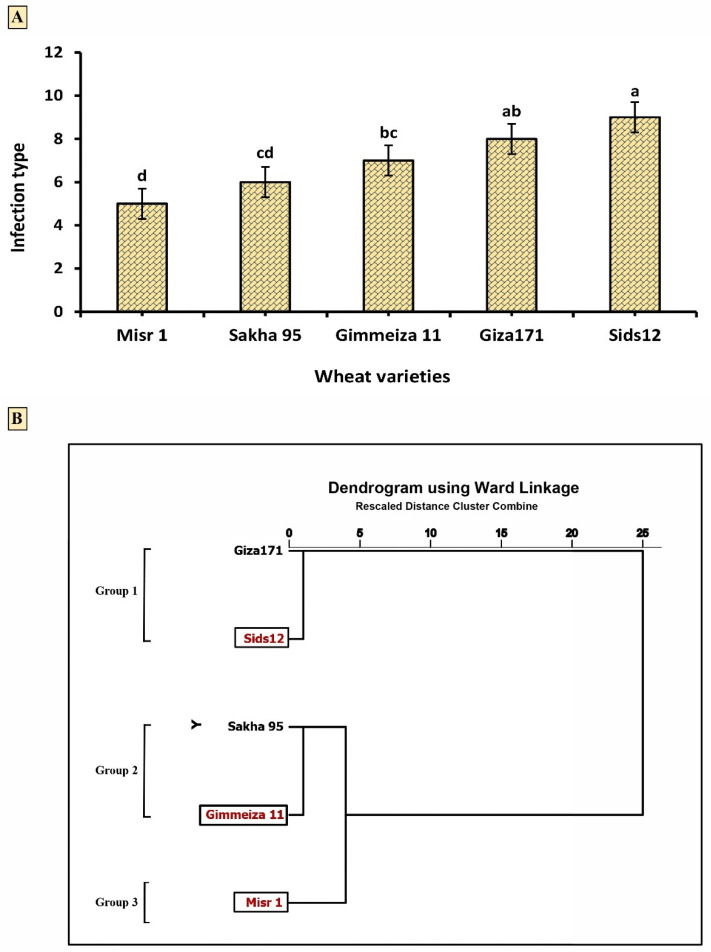
(**A**) Comparative analysis of five wheat varieties in response to stripe rust infection. The assigned letters denote the results of Duncan’s multiple range test performed *p* < 0.05. (**B**) Dendrogram of different wheat varieties based on infection type response against *Pst*.

The results indicated in Figure 3A suggested that among the five wheat varieties examined, Sids12 had the highest level of stripe rust infection, with a rating of 9.00. Giza171, Sakha95, and Gimmeiza11 all had moderate levels of stripe rust infection, with ratings of 8.00, 6.00, and 7.00, respectively. Misr1 had the lowest level of stripe rust infection, with a rating of 5.00. In addition, Figure 3B shows a dendrogram of clustering analysis of five wheat varieties based on their infection-type response against *Pst*. The analysis was performed using Ward linkage. Accordingly, the five wheat varieties can be grouped into three distinct clusters. The first cluster (Group 1) includes the varieties Giza171 and Sids12. These varieties are both very susceptible to *Pst* infection. The second cluster (Group 2) includes the varieties Sakha95 and Gimmeiza11. These varieties are both moderately susceptible to *Pst* infection. The third cluster (Group 3) includes the variety Misr1. This variety is moderately resistant to Pst infection.

**Figure 4:**
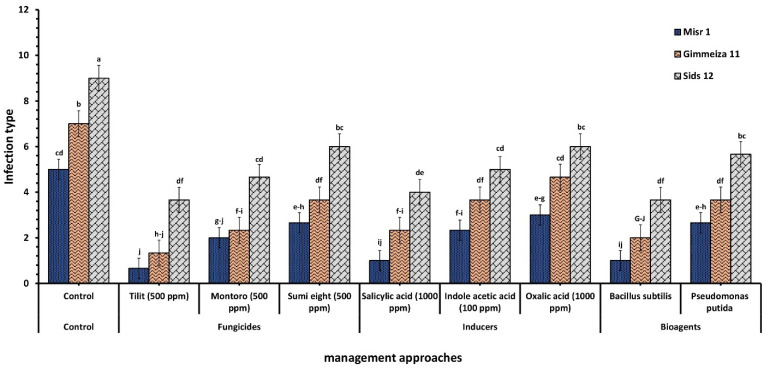
Comparative analysis of three wheat varieties (Misr1, Gimmeiza11, and Sids12) in response to stripe rust infection under three management approaches: fungicides, chemical inducers, and bioagents. Tilit 25% EC, Montoro 30% EC, and Sumi eight. The assigned letters (e.g., ‘a’, ‘b’, ‘c’) denote outcomes of Duncan’s multiple range test performed at a significance level of *p* < 0.05.

While Figure 4 shows the response of the tested varieties (Misr1, Gimmeiza11, and Sids12) to three different management approaches for stripe rust infection. The control group had the highest infection rate, with Misr1 having the lowest of the three varieties at 5.00. The infection rate varied in response to different management methods. The lowest variety upon infection was Misr1, followed by Gimmeiza11 and Sids12. Misr1 showed the lowest infection rate of (0.66) observed with Tilit 25% EC (500 ppm), followed by the infection rate of (1.00) seen with Salicylic acid (1000 ppm) and B. subtilis. Gimmeiza11 had the second-lowest infection rate in all three groups, with 1.33, 2.33, and 2.00 for Tilit, Salicylic acid, and B. subtilis, respectively. While Sids12 had the highest infection rate in all three groups with (6.00), which was observed in Sumi eight 5% EC (500 ppm), oxalic acid (1000 ppm), and *P. putida*.

### 2.3. Wheat Stripe Rust Infection Type upon Application of Three Tested Chemical Inducers

Data in Table 1 shows the impacts of different concentrations of chemical inducers, Salicylic acid, Indole acetic acid, and oxalic acid, on the SR infection type in wheat plants before and after inoculation for three wheat varieties (Misr1, Gimmeiza11 and Sids12). The stripe rust infection type of the control treatment was 5.00 for Misr1, 7.00 for Gimmeiza11, and 9.00 for Sids12, either before or after the inoculation. All treatments showed a reduction in the infection type, and it was also reduced after inoculation compared to before inoculation. Additionally, increasing the concentration of the tested inducers led to a decrease in the stripe rust infection type for all three wheat varieties.

For Salicylic acid, the results showed that the lowest infection type was recorded at 1000 ppm with 2.33, 3.66, and 5.66 (before inoculation), while 1.00, 2.33, and 4.00 (after inoculation) for Misr1, Gimmeiza11 and Sids12, respectively. For Indole acetic acid, a similar trend was observed with decreasing stripe rust infection type as the concentration increased. The highest concentration (100 ppm) showed the lowest infection type of 3.33, 4.66, and 6.66 before inoculation, while it was 2.33, 3.66, and 5.00 after the inoculation, for Misr1, Gimmeiza11, and Sids12, respectively. Before the inoculation, the infection type was 7.66 for Sids12, and after the inoculation, it was 6.00. For oxalic acid, the results showed that the stripe rust infection type decreased with increasing concentration. The highest concentration (1000 ppm) showed the lowest infection type of 3.00 after the inoculation in Misr1. Before the inoculation, the infection type was 7.66 for Sids12, and after the inoculation, it was 6.00, while it was 5.33 before inoculation and then reduced to 4.66 after inoculation for Gimmeiza11. Accordingly, Salicylic acid proved the best results in reducing the infection type among the other tested inducers, followed by Indole acetic acid, while oxalic acid had the lowest decrease in stripe rust infection type.

### 2.4. Wheat Stripe Rust Infection Type upon Application of Three Tested Fungicides

Data in (Table 2) revealed the impact of three fungicides (Tilit 25% EC, Montoro 30% EC, and Sumi eight 5% EC) at different concentrations (125 ppm, 250 ppm, and 500 ppm) on stripe rust infection type in wheat plants was tested before and after inoculation for three wheat varieties (Misr1, Gimmeiza11, and Sids12). For the wheat variety Misr1, the control treatment resulted in an infection rate of 5.00 before and after inoculation. When treated with (Tilit), the infection type decreased to 1.66 and 2.66 at 125 ppm and 250 ppm, respectively, after inoculation, while 500 ppm showed the least infection type of 0.66. For Gimmeiza11, the control treatment resulted in an infection type of 7.00 before and after inoculation. The lowest infection type was recorded at 500 ppm of (Tilit) with 3.00 before inoculation and 1.33 and after inoculation. For Sids12, the control treatment resulted in an infection type of 9.00 before and after inoculation. The infection type decreased to 3.66 after inoculation and 5.33 before inoculation.

After treatment with (Tilit), the best infection type reduction was recorded at 500 ppm with 3.66, 1.33, and 0.66 for Sids12, Gimmeiza11, and Misr1, respectively. Treatment with (Montoro) showed similar trends in infection type reduction for all three wheat varieties, with the lowest type recorded at 500 ppm for Misr1, Gimmeiza11, and Sids12 with 2.00, 2.33, and 4.66, respectively. Lastly, treatment with (Sumi 8) showed the highest infection type for all three wheat varieties. The highest reduction in infection type was recorded at 500 ppm for Misr1, Gimmeiza11, and Sids12, with 2.66, 3.66, and 6.00, respectively. Accordingly, the results showed that all three fungicides were effective in decreasing stripe rust infection in the three wheat varieties tested, with (Tilit) showing the best results and the lowest infection type; however, (Sumi 8) showed the highest records of infection type. Additionally, the concentration of 500 ppm was the best for all fungicides to achieve better reduction in the infection type. Also, results revealed that Sids12 exhibited the highest infection type, while Mis 1 was the lowest infected variety in the control.

### 2.5. Antioxidant Enzyme Activity in Wheat-Stripe Rust Interaction

#### 2.5.1. Catalase Activity

**Figure 5:**
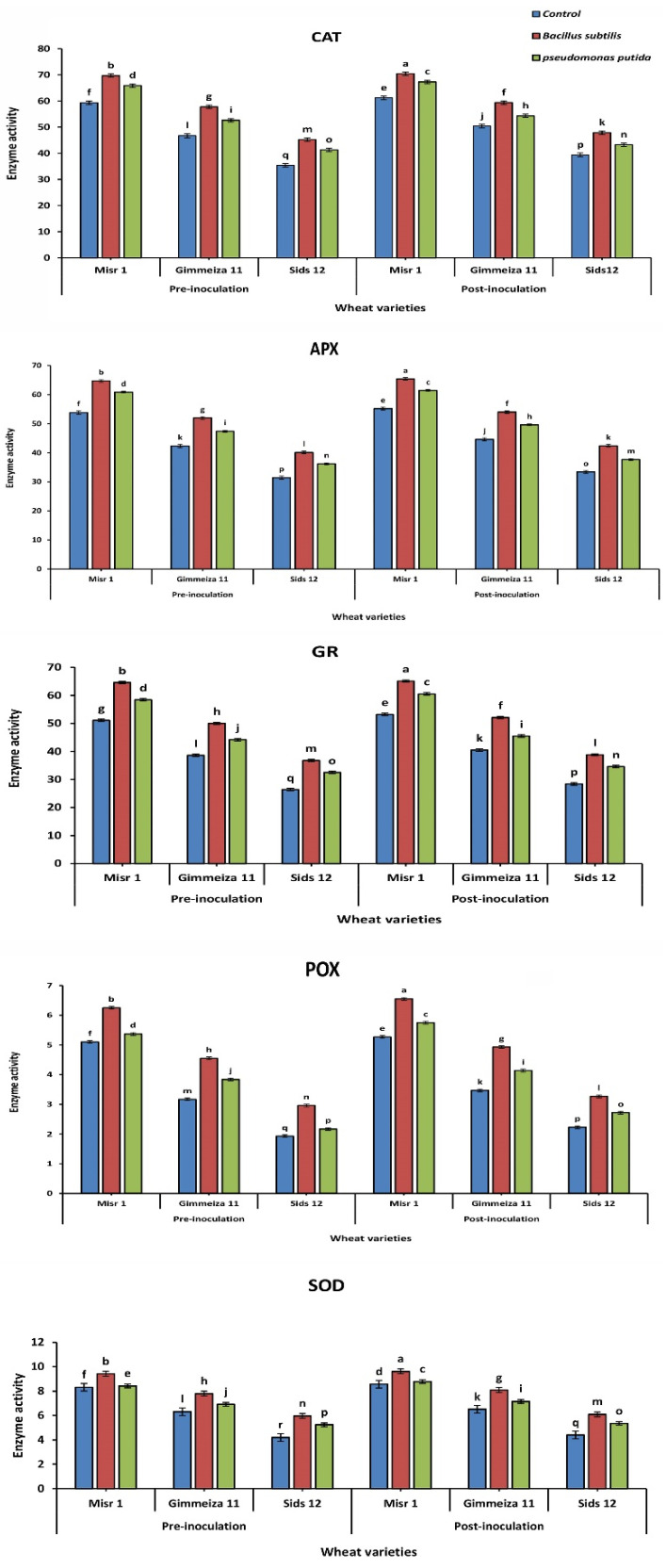
The enzymatic activities of five enzymes—CAT, APX, GR, POX, and SOD—were evaluated on three wheat varieties, namely Misr1, Gimmeiza11, and Sids12. The assessment was conducted upon treatment with two bacterial bioagents, *B. subtilis*, and *P. putida*, both before and after inoculation with the *Pst* pathogen. The letters assigned following Duncan’s analysis at a significance level of *p* < 0.05, denoting significant differences, while common letters denote nonsignificance.

As shown in (Table 3a,b and Figure 5), the activity of catalase enzyme in wheat plants infected by stripe rust was increased after treatment with the three tested fungicides. The increase in activity was concentration-dependent and differed among the three wheat varieties tested. Misr1 wheat variety showed the most notable increase in activity when treated with Tilit 25% EC at a concentration of 500 ppm after inoculation (70.6). Salicylic acid at a concentration of 1000 was the most effective inducer, increasing catalase activity in all three wheat varieties after inoculation Misr1 (70.4), Gimmeiza11 (58.5), and Sids12 (46.5). The results showed that the enzyme activity increased in all wheat varieties after inoculation with the two bacterial bioagents. Misr1 exhibited the highest increase in activity after being sprayed with *B. subtilis* (70.4), followed by *P. putida* (67.3). Similarly, the enzyme activity in Gimmeiza11 and Sids12 also increased after inoculation with both bacteria.

#### 2.5.2. Ascorbate Peroxidase (APX) Activity

The chemical inducers’ impact on APX enzyme activity is indicated in (Table 4a,b and Figure 5). All three fungicides after inoculation increased APX enzyme activity in all wheat varieties, with Tilit showing the highest after inoculation at a concentration of 500 ppm misr1 (65.5), Gimmeiza11 (54.6), and Sids12 (43.5), the lowest increased APX enzyme activity in treatments groups with Sumi8 before inoculation at a concentration of 125 ppm in all wheat varieties Misr1 (55.9), Gimmeiza11 (44.2), and Sids12 (33.1). After inoculation, the highest APX activity with salicylic acid in all varieties at a concentration of 1000 ppm misr1 (65.3), Gimmeiza11 (53.3), and Sids12 (41.1), while oxalic acid at a concentration of 250 ppm had the lowest APX activity before inoculation in all wheat varieties Misr1 (55), Gimmeiza11 (42.1), and Sids12 (31.6), regardless of treatment group. Also, the results showed that the APX enzyme activity increased in all three wheat varieties after inoculation with both endophytic bacteria. For Misr1, the enzyme activity increased (65.4) with *B. subtilis* and (61.4) with *P. putida*. Similar increases were observed for Gimmeiza11, (54) with *B. subtilis* and (49.6) with *P. putida* and Sids12, (42.4) with *B. subtilis* and (37.7) with *P. putida*.

#### 2.5.3. Glutathione Reductase (GR) Activity

Table 5a,b and Figure 5 revealed that Tilit 25% EC showed the highest increase in GR enzyme activity after inoculation in Misr1 (65.5) at a concentration of 500 ppm, while the highest increase in Gimmeiza11, (52.3) was seen at the same fungicide and concentration. For Sids12 (39.4), the highest increase was seen with Tilit 25% EC at 500 ppm. Results showed that all three wheat varieties showed increased GR enzyme activity when treated with different chemical inducers compared to the control. Salicylic acid had the highest effect on increasing GR enzyme activity after inoculation in Misr1 (64.6) at a concentration of 1000 ppm, while oxalic acid had the lowest effect before inoculation in Sids12, (27.0) at a concentration of 250 ppm. Results showed an increase in GR enzyme activity in all wheat varieties after inoculation with both endophytic bacteria Misr1, with *B. subtilis* (65.1) and with *P. putida* (60.5). Gimmeiza11, (52.1) with *B. subtilis* and (45.5) with *P. putida* and Sids12, (38.8) with *B. subtilis* and (34.6) with *P. putida*, indicating that they can be effective biocontrol agents for increasing the resistance of wheat plants to stripe rust.

#### 2.5.4. Superoxide Dismutase (SOD) Activity

Data in Table 6a,b and Figure 5 indicated that the control group showed SOD enzyme activity of 8.38, 6.31, and 4.20 in the pre-inoculation stage for Misr1, Gimmeiza11, and Sids12, respectively. After inoculation, the SOD enzyme activity increased to 8.57, 6.51, and 4.40. All three fungicides showed an increase in SOD enzyme activity for the three wheat varieties in both pre- and post-inoculation stages. Propiconazol (Tilit 25% EC) with 500 ppm concentration showed the highest increase after inoculation in Misr1 (9.93), while sumi 8 with 125 ppm showed the lowest increase in SOD enzyme activity before inoculation in Sids12 (4.77). Chemical inducers’ effect was also tested, revealing that the SOD activity was tested before and after inoculation with the inducers and compared to a control group. Salicylic acid with 1000 ppm concentration in post-inoculation showed the highest increase in SOD activity in all three varieties with (9.26, 7.76, and 5.95), followed by Indole acetic acid with 100 ppm concentration (8.83, 7.22, and 5.43), while oxalic acid with 1000 ppm concentration had a weaker effect with (8.38, 6.68 and 4.89). The SOD enzyme activity was measured before and after inoculation with bacterial bioagents. Results showed that SOD activity was increased after inoculation with both bacteria in all three wheat varieties. For example, in Misr1, the SOD enzyme activity was 9.42 before inoculation and 9.62 after inoculation with *B. subtilis*. The control group also showed an increase in SOD with 1000 ppm concentration activity after inoculation with both bacteria.

#### 2.5.5. Peroxidase (POX) Activity

As reported in (Table 7a,b and Figure 5), results showed that at 500 ppm concentration, Tilit had the greatest impact on increasing POX enzyme activity in all three wheat varieties post-inoculation, with (6.89, 5.09, and 3.31), while Montoro also had a positive effect but to a lesser extent with (6.35, 4.55, and 2.95). Sumi-8 had the least impact on increasing POX activity with (5.81, 4.01, and 2.59). Also, data showed that salicylic acid at 1000 ppm had the most significant effect post inoculation with (6.22, 4.72, and 3.22), followed by Indole acetic acid at 100 ppm with (5.77, 4.27 and 2.77), and then oxalic acid at 1000 ppm with (5.32, 3.82, and 2.32). In addition, the POX activity of all wheat varieties was higher when sprayed with *B. subtilis* post inoculation with (6.25, 4.55, and 2.96), while the lowest activity when sprayed with *P. putida* with (5.75, 4.14, and 2.72 as indicated in (Figure 5).

## 3. Discussion

### 3.1. Wheat Stripe Rust Infection Type upon Application of Two Tested Bacterial Bioagents

The results of the study showed that the application of endophytic bacteria, *B. subtilis* and *P. putida*, reduced the stripe rust infection type in all three wheat varieties. This finding is consistent with previous studies that have reported the efficacy of bacterial bioagents in controlling plant diseases, including wheat stripe rust. For example, a study conducted by [23] found that the *B. subtilis* strain (E1R-J) has reduced the disease severity of WYR in both greenhouse and field experiments. Also, many studies reported that *B. subtilis* acts as an abio-fungicide with a significant reduction in the severity of WYR disease, acting as a bio-fungicide [24,25,26,27]. In addition, *B. subtilis* strain QST 713 showed its potential for WYR control at the early growth stage under moderate disease pressure in winter wheat field trials [14,25]. *B. subtilis* exhibited inhibition of spore germination and prolonged incubation and latent periods compared to other treatments. Additionally, *B. subtilis* reduced the SR infection type, pustule length, pustule width, and number of pustules [26]. Treated leaves exhibited various abnormalities, including lysis, collapse, and shrinking of urediniospores. These observations may be attributed to the production of antibiotics by *B. subtilis*, such as bacillinbacitracin, bacillomycin, mycosubtilin, subsporin, subenolin, and subtilin [28]. These antibiotics effectively reduced disease development and minimized pustule size [26,29].

On the other hand, another study reported that *P. putida* strain JD204, isolated from wheat roots, was reported to activate resistance by the over-expression of the resistance-related genes [24]. Also, *P. putida* ASU15 was applied during pathogen inoculation; it resulted in a higher reduction in disease severity of common bean rust (69.9%) compared to its application before pathogen inoculation (54.9%) [30]. *P. putida* can produce various antibiotics, siderophores, and a small amount of hydrogen cyanide (HCN). These substances effectively inhibit the growth of *P. triticina* both in vitro and in vivo [31].

The mechanism of action of endophytic bacteria in controlling plant diseases involves several mechanisms, such as competing for nutrients and space, as well as producing antimicrobial compounds, and induction of systemic resistance in the host plant [32,33]. *B. subtilis* and *P. putida* are capable of producing a diverse array of antimicrobial compounds that effectively hinder the growth of plant pathogens, including fungi and bacteria [14,34]. In addition, these bacteria can induce systemic resistance in plants, which is a defense mechanism that enables plants to resist infection by pathogens [35]. Applying bioagents has the potential to bring about beneficial changes in the physical and mechanical integrity of cell walls, as well as adjust the physiological and biochemical responses of wheat plants. This, in turn, can boost the production of defense-related molecules that are crucial in delaying the inoculation and latent period of *P. graminis*. Furthermore, *Bacillus* spp. offer multiple methods for disease control, including the synthesis of antifungal compounds, nutrient competition, and the induction of systemic resistance [26,36].

The effectiveness of *Bacillus* spp. along with *P. fluorescens* as bio-agents in suppressing stripe rust disease in wheat plants was investigated [37]. *B. subtilis* and *B. chitinosporus* demonstrated the highest reduction in disease severity. Other studies have also supported the use of *B. subtilis* as a bio-agent for enhancing the tolerance of wheat plants under leaf rust disease stress [38]. *Pst* urediniospores and fungal hyphae engaged in competition for the entry site, while metabolites released by biological agents effectively impeded the growth of germ tubes. Moreover, the activation of PR protein genes may contribute to the development of resistance responses in inoculated plants [39]. In addition, two bioagents, *P. fluorescence* and *B. subtilis*, exhibited moderate effectiveness in controlling stripe rust, achieving reductions of 54.25% and 56.33%, respectively [40].

Overall, the results of this study suggest that the application of *B. subtilis* and *P. putida* can be an effective strategy for controlling wheat stripe rust. However, further research is needed to optimize the application methods and to evaluate the long-term effects of these bioagents on wheat growth and yield.

### 3.2. Wheat Varietal Reaction in Response to Stripe Rust Infection

Data in Figure 3A shed light on the susceptibility of five varieties to yellow rust disease. Misr1 had the lowest level of infection; however, Sids12 had the highest level of stripe rust infection among the five wheat varieties examined. Giza171, Gimmeiza11, and Sakha 95 had moderate levels of infection. This suggests that Misr1 is relatively resistant to stripe rust compared to the other varieties evaluated. In addition, results in (Figure 4) evaluated the efficacy of three management approaches on three selected wheat varieties to stripe rust infection. The infection rate varied among the three management approaches, with Misr1 showing the lowest infection rate in response to Tilit 25% EC (500 ppm), Salicylic acid (1000 ppm), and *B. subtilis*. Gimmeiza11 had the second-lowest infection rate in all three groups, while Sids12 had the highest infection rate in all three groups.

Prior investigations have evaluated different wheat genotypes for their resistance potential. For example, In Egypt, Giza 168, Sakha-61, Sakha-93, Gimmeiza7, and Gimmeiza9 demonstrated satisfactory resistance levels in a two-year study [41]. However, Omara et al. [42] revealed that widely grown cultivars like Gemmeiza11 and Sids12 exhibited susceptibility to stripe rust under Egyptian field conditions. This contradicts their widespread cultivation across the country. Also, [43] stated that certain wheat cultivars, namely Giza168, Sakha93, Sids12, Gemmieza7, Gimmieza9, Gimmieza11, Sids1, and Sids13, were found to be susceptible to stripe rust. However, the cultivars Misr-1 and Misr-2 demonstrated resistance to the disease. Similarly, previous studies reported that Sids12 had the highest disease progression and yield loss. Also, Gimmeiza11 exhibited significant grain yield losses of 64.20% due to wheat stripe rust [6]. In contrast, our findings indicate that Misr1 exhibited promising outcomes in reducing wheat stripe rust infection. However, these results contradict a study conducted by [6], which classified Misr1 as a susceptible cultivar. The variation in response among the tested wheat cultivars can be attributed to genetic diversity, environmental conditions, and the emergence of aggressive rust pathogen races [6]. It is important to note that these results are specific to the conditions and period of the study. Therefore, caution should be exercised when generalizing these findings to other locations or time periods.

In addition, the susceptible wheat cultivar showed extensive colonization and high spore production when infected with *P. striiformis*, along with intercellular hyphae and haustoria. In contrast, the resistant cultivar exhibited limited and abnormal haustoria and hyphal development. Resistant wheat leaves had small, shriveled spores with a low spore count per sorus, while susceptible wheat leaves had numerous sori with a high spore quantity [44]. The reasons for the different varietal reactions to stripe rust infection could be attributed to genetic differences in the resistance/susceptibility of the wheat varieties [6,45,46,47]. Therefore, genetic factors play a significant role in the phenotypic variations observed in the current study. The most effective and environmentally safe approach for managing wheat stripe rust is through host-genetic resistance or the cultivation of resistant wheat cultivars [45]. Moreover, the Yr gene, responsible for yellow rust resistance, regulates defense-related genes. Transcriptomics research has identified multiple genes involved in seedling-stage resistance [39,48]. Previous studies have reported the existence of different resistance genes in wheat that confer resistance to SR, such as Yr5, Yr10, and Yr15 [46,49].

Interestingly, the national wheat breeding program in Egypt relies on resistant genotypes from CIMMYT, ICARDA, and local sources. Nonetheless, in recent years, numerous commercial and recommended wheat cultivars have shown varying degrees of susceptibility to stripe rust nationwide [42]. Overall, the search for new sources of resistance is crucial to enhance stripe rust resistance in local breeding materials and reduce the need for extensive disease management efforts [47].

### 3.3. Wheat Stripe Rust Infection Type upon Application of Three Tested Chemical Inducers

The results showed that all tested inducers led to a reduction in the infection type, with higher concentrations of the inducers leading to a greater decrease in infection type. SA stimulates growth, enhances yield, and counters the pathogenic effects by inducing host resistance [50]. Previous studies have also explored the use of chemical inducers to control stripe rust infection in wheat. For example, Cheng et al. [51] found that salicylic acid was involved in the non-host resistance (NHR) of Arabidopsis against wheat stripe rust. Another study by [52] found that the application of Salicylic acid reduced stripe rust severity in wheat plants as measured by AUDPC (area under production curve). Since defense genes were activated after salicylic acid treatments compared to controls [53], it was confirmed that the application of SA in wheat can activate PR-1 and PR-2 [14]. On the other hand, another study by [54] showed that Indole acetic acid treatment at 100 µgmL^−1^ reduced stripe rust infection and induced rust resistance in wheat cv. Tamuz-2. Similarly, previous researchers indicated that indole acetic acid plays a significant role in inducing wheat defense mechanisms and reducing the severity of wheat stripe rust disease [18,52]. Our findings align with previous studies, providing further support for the effectiveness of SA and IAA in reducing the SR infection type in wheat plants.

The reason for the effectiveness of salicylic acid and indole acetic acid in reducing stripe rust infection type may be due to their ability to induce plant defense responses against pathogens [14]. Salicylic acid has a main role in systemic acquired resistance (SAR), a plant defense mechanism against pathogens, while Indole acetic acid has been shown to induce the expression of genes involved in plant defense responses [11,18,55]. In contrast, the current study found that oxalic acid had the lowest decrease in stripe rust infection type. Previous studies have also shown mixed results with the use of oxalic acid to control fungal pathogens in plants. For example, a study by [56] found that oxalic acid had no significant effect on powdery mildew infection in wheat plants. However, ref. [57] recorded the maximum disease severity using oxalic acid compared with other chemical inducers on French bean rust under pot conditions with 35.33%.The lack of effectiveness of oxalic acid in the current study may be due to differences in the pathogen, plant species, or experimental conditions. Overall, the findings of the current study suggest that salicylic acid and indole acetic acid can be effective in reducing stripe rust infection type in wheat plants, while the effectiveness of oxalic acid may be limited.

### 3.4. Wheat Stripe Rust Infection Type upon Application of Three Tested Fungicides

Applying fungicides for the management of plant diseases is a well-established practice in agriculture. Several previous researchers have examined the impact of different fungicides against WYR. The present study provides further evidence for the effectiveness of Tilit and Montoro in controlling stripe rust in wheat while also highlighting the limitations of Sumi 8 in this regard. The use of fungicides at a concentration of 500 ppm was found to be highly effective in minimizing the infection type of stripe rust in the tested wheat varieties. The results of the study also emphasize the importance of selecting appropriate fungicides for controlling plant diseases based on their efficacy and the genetic characteristics of the crop.

The most employed fungicides for managing stripe rust disease are demethylation inhibitors (DM1; triazole) class [12,58,59,60]. These fungicides target specific enzymes or vital metabolic pathways within fungi [61]. Notably, propiconazole, Difenoconazole, and Diniconazole are prominent triazole fungicides, serving as the active ingredients in the tested fungicides (Table 8). The triazole fungicides hinder the function of the 14α-demethylase enzyme, which is encoded by the Cyp51 genes. This inhibition prevents the synthesis of ergosterol, a crucial component for preserving the integrity of the fungal cell membrane. Additionally, these fungicides impede spore formation and hinder the growth of fungi at the stage of first haustoria formation, causing stunted development [62,63,64,65,66]. The triazole-based formulations are highly popular plant protection products due to their effective protective and curative properties, best applied during the early stages of disease development [67,68,69].

Propiconazole, registered as Tilt, has been utilized for over two decades [70,71]. Tilt operates systemically, offering both protective and curative capabilities, primarily inhibiting fungal growth [72]. A study by [73] found that Tilit was effective in controlling stripe rust in wheat at a concentration of 250 ppm. Similarly, another study by [74] showed that the spray of Tilt 0.1 percent drastically decreased the stripe rust in all the assessed varieties. Ref. [75] supported the high efficacy of propiconazole fungicides such as Tilt 25EC, which is particularly effective against leaf rust and yellow rust. Also, propiconazole 25EC demonstrated significant effectiveness in reducing the severity of yellow rust by 80.2% [40]. When Tilt was used at the recommended concentration, it effectively suppressed urediniospore germination in all tested isolates. However, lowering the fungicide concentrations resulted in varying germination rates among the fungal isolates [76].

On the other hand, the inclusion of diverse, active ingredients in new fungicides further complicates the categorization of their protective roles [12]. Montoro is a systemic fungicide that has two active substances, Difenoconazole and Propiconazole, which give a wide double effect on many fungal diseases such as WYR, as indicated in the current study. Zhang et al. [69] suggested that the use of difenoconazole and propiconazole on wheat is considered to be safe under the Good Agricultural Practices (GAP) in the Chinese fields, and the main factors for pesticide residue in crops are application times, rates, and pre-harvest intervals. Previous studies have also shown the effectiveness of foliar spraying of difenconazole, propiconazole, and tebuconazole against this disease [71,77,78]. Difenoconazole is one of the most widely used (DMI; triazole) fungicides for reducing the severity of various plant diseases, such as stripe rust [68,72]. Moreover, the combination of propiconazole and azoxystrobin application has emerged as the optimal choice in controlling WYR, as these chemicals possess two different modes of action [72,79,80,81].

In turn, Sumi 8 has shown a reduction in the disease severity; however, it revealed the lowest results among the tested fungicides. Similarly, previous studies indicated that Sumi-8 prevented spore germination of *P. triticina* [26,82]. Overall, our results provide further evidence for the effectiveness of Tilit and Montoro fungicides in controlling WYR. The findings underscore the importance of selecting appropriate fungicides based on their efficacy, concentration, and genetic characteristics of the crop.

### 3.5. Antioxidant Enzyme Activity in Wheat-Stripe Rust Interaction

The study investigated the effects of three fungicides, chemical inducers, and two bioagents on the activity of antioxidant enzymes in three wheat varieties. The observed increases in antioxidant enzyme activity in wheat plants infected with stripe rust and treated with various inducers and fungicides can be attributed to the plants’ defense response against the pathogen [83]. Catalase and ascorbate peroxidase are important enzymes that scavenge reactive oxygen species (ROS) and protect plant cells from oxidative damage during pathogen attacks [84]. Catalase (CAT) is an essential oxidative enzyme that boosts host resistance against plant pathogens by limiting pathogen spread and activating defense genes [85]. Increased CAT activity helps prevent the accumulation of hydrogen peroxide levels, which can be detrimental to cells, and serves as a secondary signal for defense gene expression and SAR [85,86]. Similarly, glutathione reductase (GR) has a key role in maintaining the cellular redox balance, which is essential for plant resistance against pathogens [87]. Peroxidase (POX) is another enzyme involved in plant defense responses and serves as an indicator of disease resistance [88,89]. POX plays a direct role in the synthesis of lignin, enhancing the ability of protected tissues to resist pathogens [89]. Superoxide dismutase and peroxidase enzymes also have been shown to play a role in plant resistance against pathogens by removing ROS and activating defense-related genes [90,91]. The induction of these antioxidant enzymes by various chemical inducers and fungicides may contribute to the enhanced resistance of wheat plants against stripe rust infection.

Interestingly, Pathogen infection leads to increased reactive oxygen species (ROS), causing oxidative stress [92]. Plants respond by activating antioxidant enzymes (superoxide dismutase (SOD), catalase CAT, glutathione reductase, peroxidase POX, and ascorbate peroxidase (AP) and non-enzymatic antioxidants (ascorbate, phenolic compounds, and carotenoids) [93]. Susceptible wheat cultivars show reduced activity of catalase (CAT) and peroxidase (POX) compared to resistant cultivars [43]. CAT and POX play a crucial role in defending resistant wheat cultivars by counteracting elevated ROS levels caused by pathogens [26,38,94].

In treatments with *B. subtilis*, the activities of (CAT), (POX), and (PPO) were significantly increased, resulting in reduced infection [26]. Aboulila [18] investigated the induction of systemic acquired resistance (SAR) in susceptible wheat (Sids-12) against (*Pst*) using five phytohormones, including (SA), abscisic acid (ABA), indole 3-butyric acid (IBA), (IAA) and naphthaleneacetic acid (NAA). Overexpression of PR protein genes played a crucial role in activating the host defense mechanism. Phytohormones as chemical inducers offer safer and more efficient control of wheat stripe rust, reducing infection and inducing resistance. Phytohormones have a significant role in managing plant diseases by directly reducing fungal germination and development or indirectly promoting plant defense systems [95]. In the Sids-12 wheat genotype, the activation of resistance genes was observed after treatment with IAA and inoculation with *Pst* spores. The expression of PR-4 was highly induced along with PR-1 at all-time points and concentrations of IAA, indicating a significant increase in endochitinase production [18]. On the other hand, exposure to difenoconazole was found to increase the activities of antioxidant enzymes such as superoxide dismutase (SOD), catalase (CAT), guaiacol peroxidase (G-POD), and ascorbate peroxidase (APX) in both the roots and leaves of wheat seedlings [96]. This exposure also led to an increase in the production of (ROS) such as O_2_^•−^ and H_2_O_2_, as well as malondialdehyde (MDA) in plant leaf cells [96]. To repair the damage caused by ROS, plants have developed a complex enzymatic antioxidant system, including SOD, CAT, G-POD, (GR), and APX, which efficiently maintains the redox balance in plant cells by scavenging excessive ROS [96,97].

Comparing resistant and susceptible cultivars, it was observed that resistant cultivars exhibited lower rust severity and slower disease progression. Susceptible cultivars, on the other hand, showed the accumulation of (ROS) such as (O_2_^•−^) and (H_2_O_2_). In contrast, resistant cultivars demonstrated higher activity of catalase (CAT) and peroxidase (POX), along with increased chlorophyll levels [43,98]. Histological analysis revealed inhibited haustoria and hyphae in the resistant cultivar (Misr-1), while the susceptible cultivar (Sids-12) displayed abundant intercellular hyphae and haustoria [43].

The *Yr18* resistant gene was found to be overexpressed in resistant cultivars, leading to higher levels of reactive oxygen species (ROS) accumulation, specifically superoxide (O_2_^•−^) and hydrogen peroxide (H_2_O_2_), and lower activities of catalase (CAT), peroxidase (POX), and polyphenol oxidase (PPO) [44]. These findings were also supported by [99]. The Yr18/Lr34/Pm38 locus provides partial and long-lasting adult plant resistance (APR) against leaf rust, stripe rust, and powdery mildew in wheat [100]. It can be concluded that the resistant wheat cultivars exhibited suppressed disease severity and symptoms due to the presence of the Yr18 resistant gene and the accumulation of ROS, which potentially resulted in decreased enzyme activities and electrolyte leakage compared to susceptible cultivars [44].

The activation of PR protein genes is believed to contribute to the resistance response in plants upon pathogen inoculation [39]. Among these genes, PR1 is associated with pathogen-induced systemic acquired resistance and is considered a marker for the salicylic acid (SA) pathway, which plays a vital role in enhancing plant defense [101]. (Elsharkawy et al., 2013). Another important protein, PR2, known as β-1, 3-glucanase, is involved in wheat resistance controlled by various Yr genes. β-1, 3-glucanases regulate callose deposition and can break down fungal cell wall glucans, activating host defense mechanisms [102]. PR3 and PR4 are endochitinases that target chitin, a major component of fungal cell walls, and their activity is crucial for wheat’s defense against yellow rust [48,71]. The activation of pathogenesis-related protein genes, including PR1, PR2, PR3, and PR4, may play a role in reducing yellow rust disease severity [39].

## 4. Materials and Methods

### 4.1. Seedling and Varietal Reaction

Seedling testing is more frequently employed in controlled greenhouse environments, as it only requires a limited area and a few weeks. For seedling experiments, plants with one to two leaves that are seven to fourteen days old were employed. To conduct the experiment, we utilized seeds from five different wheat cultivars: Misr1, Sakha 95, Gimmeiza11, Giza 171, and Sids12. Each seed was planted individually in a pot measuring 7 cm in diameter and 10 cm in height. The pots were filled with a mixture of compost and soil in a 2:1 ratio by volume. To ensure optimal growth conditions, the plants were cultivated in a greenhouse that was free from any rust contamination. The cultivation process followed a well-established procedure, as described previously [103]. Three varieties were selected for in-depth analysis, including Misr1, Gimmeiza11, and sids12.

### 4.2. Inoculation of Pst

**Figure 6:**
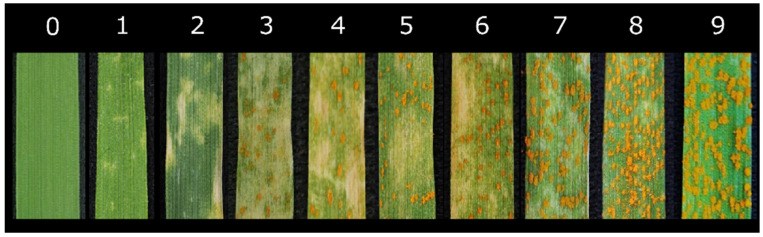
Infection type scale of stripe rust on wheat seedlings showing different proportions of sporulation and necrosis, as described by Chen [22].

Around 10 days after the initial planting, the seedlings were subjected to inoculation when the first leaves had fully grown and the second leaves had approximately halfway emerged. The selection of the most virulent *Pst* strain for further investigation was made from a group of 34 isolates based on their virulence phenotypes on wheat differential hosts. A mixture of fresh urediniospores and talc was applied at a ratio of 1:20. To facilitate the growth of the inoculated plants. They were placed in a dew chamber for incubation. Subsequently, they were transferred to a growth chamber with specific temperature and light conditions. Initially, the plants were kept in darkness at a temperature range of 10 to 13 °C for 24 h. Then, they were moved to a growth chamber with an 8-h dark cycle followed by a 16-h light cycle. With three replications of each variety. About 15 to 20 days after inoculation, symptoms begin to appear, and study their effects on infection type, as documented by [104]. Infection-type data were recorded 20 days after *Pst* inoculation based on a 0 to 9 scale, as shown in Table 9, as well as Figure 6 according to [22].

### 4.3. Disease Management

Three methods were used along with varietal reactions to control stripe rust (biological control, induced resistance, and fungicides) for three wheat cultivars (Misr1, Gimmeiza11, and Sids12) in seedling stage under controlled greenhouse conditions. All management approaches were effectively implemented 24 h before and after the *Pst* inoculation.

#### 4.3.1. Biological Control

The bio-control agents employed in this study were *Bacillus subtilis* (accession number LC599401.1) and *Pseudomonas putida* PCL1760. To prepare the nutrient media, it was first formulated and then subjected to autoclaving at 121 °C and 15 psi for a duration of 15 min. Following inoculation, the media were maintained at a temperature of 37 °C until it was manually harvested. After harvesting, the culture was stored in distilled water at a temperature of 4 °C. For the bacterial suspension, the bacterial strain was combined with 0.25 mL of water in a total volume of 1500 mL. This suspension contained 15 g of dextrose, 0.25 g of chitosan, and 0.25 g of salicylic acid. The ingredients were thoroughly mixed to form a homogeneous suspension, as described in reference [11]. The total number of pots was divided into two groups: the first group underwent pre-inoculation with *Pst*, while the second group underwent post-inoculation treatment. Each group was separately sprayed with various materials to assess their effects on infection type. For each treatment, three replicates were utilized.

#### 4.3.2. Chemical Inducers

Chemical inducers used were Indole Acetic Acid (IAA) (25, 50, and 100 ppm), Oxalic Acid (OA) (250, 500, and 1000 ppm), and Salicylic Acid (SA) (250, 500, and 1000 ppm). Stock solutions from (IAA) at the concentration of 100 ppm were prepared by dissolving 1 g of (IAA) in 10 mL ethyl alcohol and then diluted with dH_2_O to reach the required volume of 1000 mL. Different required concentrations were obtained by diluting the stock solution accordingly. For both OA and SA, the Stock solution of 1000 ppm was produced by dissolving a weight of 1 gm (OA or SA) with distilled water, then completed to 1000 mL. Required concentrations were prepared by diluting the stock solution.

#### 4.3.3. Chemical Fungicides

Seeds of three previously mentioned wheat cultivars were sown in pots (20 seeds for each 7 cm in diameter pots). One week later, seedlings were thinned to 10 wheat seedlings per pot. Chemical control used three systemic fungicides, i.e., Tilt, Montoro, and Sumi-8 (Table 4), was carried out as foliar spray application under controlled greenhouse conditions using different concentrations (125, 250, and 500 ppm) for each fungicide.

### 4.4. Determination of Antioxidants Activities

Extraction was carried out in accordance with [105] for enzyme activity. Spectrophotometric analysis was employed to evaluate the activity of the catalase (CAT) enzyme in accordance with [106]. The calculation of peroxidase (POD) activity was based on the methods described by [107]. Spectrophotochemical analysis was used to measure ascorbate peroxidase (APX) in accordance with [108]. The activity of superoxide dismutase (SOD) was determined by measuring the decrease in absorbance of the superoxide-nitro blue tetrazolium complex, as stated by [109]. Three absorbance measurements at 340 nm were used to track the oxidation of NADPH before measuring the glutathione reductase (GR) activity [110].

### 4.5. Statistical Analysis

Analysis of variance (ANOVA) was accomplished utilizing the software Statistix 8.1 to evaluate the significance of the different treatments. To determine if there were any significant differences, treatments were compared using the LSD (Least Significant Difference) test at a significance level of (*p* < 0.05).

## 5. Conclusions

The study aimed to evaluate the effectiveness of fungicides, chemical inducers, and bacterial bioagents in reducing wheat stripe rust infection in three wheat varieties. Results showed that Tilit fungicide had the best performance in reducing infection type, followed by Montoro and Sumi 8. Salicylic acid was the most effective chemical inducer, with Indole acetic acid also showing positive results. In addition, the bacterial bioagent *B subtilis* has significantly reduced infection type. The study revealed that the activity of antioxidant enzymes CAT, APX, GR, SOD, and POX, in infected wheat plants can be modulated by fungicides, chemical inducers, and bacterial bioagents. The study demonstrated varying effectiveness in managing stripe rust depending on factors such as wheat variety, inducer type, concentration, and bacterial species used. The research highlights the significant impact of wheat variety selection on the efficacy of applied management approaches, emphasizing the importance of using resistant wheat varieties and implementing effective strategies like bioagents and fungicides to control stripe rust effectively. All tested fungicide alternatives, including chemical inducers, bioagents, and resistant varieties, provide a sustainable and environmentally friendly method to induce SAR in plants, effectively defending against stripe rust disease. Future studies should focus on understanding the genetic basis of resistance and developing sustainable management approaches. Overall, the study highlights the potential of fungicides, chemical inducers, and bioagents in enhancing wheat’s antioxidant system and inducing resistance against stripe rust.

## Figures and Tables

**Table 1:** Pre- and post-inoculation assessment of chemical inducers salicylic acid, indole acetic acid, and oxalic acid on controlling stripe rust infection in three wheat varieties (Misr1, Gimmeiza11, and Sids12).

Chemical Inducers	Con. ppm	Pre-Inoculation			Post-Inoculation		
		Misr1	Gimmeiza11	Sids12	Misr1	Gimmeiza11	Sids12
Control	0	5.00 ^j–l^	7.00 ^d–f^	9.00 ^a^	5.00 ^e–h^	7.00 ^b,c^	9.00 ^a^
Salicylic acid	250	3.66 ^n–p^	4.66 ^k–m^	6.66 ^e–g^	2.33 ^m,n^	3.33 ^j–m^	5.33 ^d–g^
	500	3.00 ^p,q^	4.33 ^l–n^	6.33 ^f–h^	1.66 ^n,o^	3.00 ^k–m^	5.00 ^e–h^
	1000	2.33 ^q^	3.66 ^n–p^	5.66 ^h–j^	1.00 ^o,p^	2.33 ^m,n^	4.00 ^h–k^
Indole acetic acid	25	4.00 ^m–o^	5.33 ^i–k^	7.66 ^c,d^	3.00 ^k–m^	4.33 ^g–j^	6.00 ^c–e^
	50	3.66 ^n–p^	5.00 ^j–l^	7.33 ^c–e^	2.66 ^l–n^	4.00 ^h–k^	5.66 ^d–f^
	100	3.33 ^o,p^	4.66 ^k–m^	6.66 ^e–g^	2.33 ^m,n^	3.66 ^i–l^	5.00 ^e–h^
oxalic acid	250	4.66 ^k–m^	6.33 ^f–h^	8.66 ^a,b^	4.00 ^h–k^	5.33 ^d–g^	7.33 ^b^
	500	4.33 ^l–n^	6.00 ^g–i^	8.00 ^b,c^	3.66 ^i–l^	5.00 ^e–h^	6.33 ^b–d^
	1000	3.66 ^n–p^	5.33 ^i–k^	7.66 ^c,d^	3.00 ^k–m^	4.66 ^f–i^	6.00 ^c–e^
L.S.D.							
Varieties		0.273			0.326		
Concentration		0.316			0.377		
Inducers		0.273			0.326		
Varieties × Concentration		0.547			0.653		
Varieties × Inducers		0.474			0.566		
Concentration × Inducers		0.547			0.653		
Varieties × Concentration × inducers		0.948			1.132		

The letters assigned (e.g., ‘a’, ‘b’, ‘c’) correspond to the results of Duncan’s multiple range test conducted at a significance level of *p* < 0.05. Different letters denote significant differences between treatments, while similar letters indicate non-significance. × refers to the interaction between variables.

**Table 2:** Effect of fungicides Tilit 25% EC, Montoro 30% EC, and Sumi eight 5% EC on controlling stripe rust infection in three wheat varieties (Misr1, Gimmeiza11, and Sids12): Pre- and post-inoculation analysis.

Tested Fungicides	Con. ppm	Pre-Inoculation			Post-Inoculation		
		Misr1	Gimmeiza11	Sids12	Misr1	Gimmeiza11	Sids12
Control	0	5.00 ^g–i^	7.00 ^c–e^	9.00 ^a^	5.00 ^d–f^	7.00 ^b^	9.00 ^a^
Tilit	125	3.00 ^l–n^	4.00 ^i–l^	6.33 ^d–f^	1.66 ^m,o^	2.66 ^j–m^	4.33 ^e–h^
	250	2.66 ^m,n^	3.33 ^k–n^	6.00 ^e–g^	1.33 ^n,o^	2.00 ^l–n^	4.00 ^f–i^
	500	2.33 ^n^	3.00 ^l–n^	5.33 ^f–h^	0.66 ^o^	1.33 ^n,o^	3.66 ^g–j^
Montoro	125	3.66 ^j–l^	5.00 ^g–i^	7.33 ^b–d^	2.66 ^j–m^	3.33 ^h–k^	5.33 ^c–e^
	250	3.33 ^k–n^	4.33 ^h–k^	7.00 ^c–e^	2.33 ^k–n^	3.00 ^i–l^	5.00 ^d–f^
	500	3.00 ^l–n^	4.00 ^i–l^	6.33 ^d–f^	2.00 ^l–n^	2.33 ^k–n^	4.66 ^e–g^
Sumi 8	125	4.66 ^h–j^	6.33 ^d–f^	8.33 ^a,b^	3.33 ^h–k^	4.66 ^e–g^	6.66 ^c^
	250	4.00 ^i–l^	5.33 ^f–h^	8.00 ^a–c^	3.00 ^i–l^	4.00 ^f–i^	6.33 ^b,c^
	500	3.66 ^j–l^	4.66 ^h–j^	7.33 ^b–d^	2.66 ^j–m^	3.66 ^g–j^	6.00 ^b–d^
L.S.D.							
Varieties		0.368			0.289		
Concent.		0.425			0.334		
Fungicides		0.368			0.289		
Varieties × Concent.		0.736			0.579		
Varieties × Fungicides		0.638			0.501		
Concent. × Fungicide		0.736			0.579		
Varieties × Concent. × Fungicides		1.276			1.003		

The letters assigned (e.g., ‘a’, ‘b’, ‘c’) correspond to the results of Duncan’s multiple range test conducted at a significance level of *p* < 0.05. Dissimilar letters denote significant differences between treatments, while similar letters indicate non-significance. × refers to the interaction between variables.

**Table 3:** (**a**) sCatalase enzyme activity on wheat plants infected by stripe rust before (pre-) and after (post-) inoculation with some fungicide (Tilit 25% EC, Montoro 30% EC, and Sumi eight 5% EC) for three wheat varieties (Misr1, Gimmeiza11, and Sids12). (**b**) Effect of chemical inducers (salicylic acid, indole acetic acid, and oxalic acid) on catalase activity in three wheat varieties (Misr1, Gimmeiza11, and Sids12) pre- and post-inoculation with stripe rust.

**(a)**							
**Fungicides**	**Concentration (ppm)**	**Pre-Inoculation**			**Post-Inoculation**		
		**Misr1**	**Gimmeiza11**	**Sids12**	**Misr1**	**Gimmeiza11**	**Sids12**
Control	0	59.5 ^h^	46.4 ^r^	35.4 ^z^	61.5 ^j^	50.4 ^q^	39.4 ^x^
Tilit	125	67.4 ^b^	55.7 ^k^	43.4 ^t^	68.7 ^c^	58.4 ^l^	47.4 ^s^
	250	69.2 ^a^	57.6 ^j^	45.1 ^s^	70 ^b^	60.1 ^k^	49.1 ^r^
	500	69.7 ^a^	58.5 ^i^	45.6 ^s^	70.6 ^a^	60.5 ^k^	49.6 ^r^
Montoro	125	63.9 ^e^	52.4 ^n^	40.2 ^v^	65.8 ^f^	55.1 ^n^	44.2 ^u^
	250	65.4 ^d^	53.6 ^m^	41.9 ^u^	67.1 ^e^	56.9 ^m^	46.2 ^t^
	500	66.5 ^c^	54.5 ^l^	42.3 ^u^	67.7 ^d^	57.3 ^m^	46.3 ^t^
Sumi 8	125	60.4 ^g^	48.4 ^q^	36.9 ^y^	62.9 ^i^	51.9 ^p^	40.9 ^w^
	250	62.2 ^f^	49.6 ^p^	38.3 ^x^	64.2 ^h^	53.6 ^o^	42.7 ^v^
	500	62.7 ^f^	50.8 ^o^	39.1 ^w^	64.9 ^g^	54.1 ^o^	43.1 ^v^
L.S.D.							
Varieties		0.223			0.423		
Fungicides		0.376			0.257		
varieties × Fungicides		0.652			0.590		
**(b)**							
**Chemical Inducers**	**Concentration (ppm)**	**Pre-Inoculation**			**Post-Inoculation**		
		**Misr1**	**Gimmeiza11**	**Sids12**	**Misr1**	**Gimmeiza11**	**Sids12**
Control	0	59.0 ^i^	45.6 ^s^	35 ^z^	60.2 ^g^	48.0 ^n^	37.1 ^u^
Salicylic acid	250	67.2 ^c^	55.7 ^l^	43 ^u^	68.3 ^b^	56.4 ^i^	44.4 ^p^
	500	69.6 ^b^	56.2 ^k^	43.4 ^u^	70.0 ^a^	58.1 ^h^	44.8 ^p^
	1000	69.6 ^a^	57.4 ^j^	44.5 ^t^	70.4 ^a^	58.5 ^h^	46.5 ^o^
Indole acetic acid	25	63.7 ^e^	52.2 ^o^	39.2 ^w^	65.9 ^d^	53.9 ^k^	41.2 ^r^
	50	65.9 ^d^	52.6 ^n^	41.3 ^v^	66.3 ^d^	54.3 ^k^	43.3 ^q^
	100	66.1 ^d^	53.8 ^m^	41.3 ^v^	67.3 ^c^	55.3 ^j^	43.3 ^q^
Oxalic acid	250	60.2 ^h^	47.8 ^r^	36.1 ^y^	62.2 ^f^	50.1 ^m^	38.1 ^t^
	500	62.0 ^g^	49.7 ^q^	37.8 ^x^	63.9 ^e^	51.8 ^l^	38.5 ^t^
	1000	62.5 ^f^	50.2 ^p^	38.2 ^x^	64.2 ^e^	52.2 ^l^	40.2 ^s^
L.S.D.							
Varieties		0.114			0.124		
Inducers		0.198			0.215		
Varieties × Inducers		0.396			0.431		

The letters assigned (e.g., ‘a’, ‘b’, ‘c’) correspond to the results of Duncan’s multiple range test conducted at a significance level of *p* < 0.05. Different letters denote significant differences between treatments, while similar letters indicate non-significance. × refers to the interaction between variables.

**Table 4:** (**a**) APX enzyme activity on wheat plants infected by stripe rust before (pre-) and after (post-) inoculation with some fungicide (Tilit 25% EC, Montoro 30% EC, and Sumi eight 5% EC) for three wheat varieties (Misr1, Gimmeiza11 and Sids12). (**b**) Induction of APX activity in stripe rust-infected wheat plants by salicylic acid, indole acetic acid, and oxalic acid treatment in different wheat varieties.

**(a)**							
**Fungicides**	**Concentration (ppm)**	**Pre-Inoculation**			**Post-Inoculation**		
		**Misr1**	**Gimmeiza11**	**Sids12**	**Misr1**	**Gimmeiza11**	**Sids12**
Control	0	53.8 ^j^	41.6 ^r^	31.4 ^y^	55.5 ^i^	44.6 ^q^	33.4 ^y^
Tilit	125	63.1 ^c^	51.2 ^l^	39.2 ^t^	64.3 ^b^	53 ^k^	42.3 ^s^
	250	63.6 ^b^	51.4 ^l^	39.5 ^t^	64.4 ^b^	53.4 ^k^	42.4 ^s^
	500	64.8 ^a^	52.5 ^k^	40.6 ^s^	65.5 ^a^	54.6 ^j^	43.5 ^r^
Montoro	125	59.5 ^f^	47.3 ^o^	36.1 ^v^	60.3 ^e^	49.7 ^m^	38.6 ^u^
	250	60 ^e^	47.9 ^n^	36.5 ^v^	61.4 ^d^	50.1 ^m^	39.1 ^u^
	500	61.2 ^d^	49 ^m^	37.5 ^u^	62.2 ^c^	51.2 ^l^	40.2 ^t^
Sumi 8	125	55.9 ^i^	44.2 ^q^	33.1 ^x^	57 ^h^	46 ^p^	35 ^x^
	250	56.4 ^h^	44.3 ^q^	33.4 ^x^	57.7 ^g^	46.8 ^o^	35.8 ^w^
	500	57.6 ^g^	45.5 ^p^	34.4 ^w^	58.9 ^f^	47.9 ^n^	36.9 ^v^
L.S.D.							
Varieties		0.119			0.185		
Fungicides		0.272			0.271		
Varieties × Fungicides		0.461			0.479		
**(b)**							
**Chemical Inducers**	**Concentration (ppm)**	**Pre-Inoculation**			**Post-Inoculation**		
		**Misr1**	**Gimmeiza11**	**Sids12**	**Misr1**	**Gimmeiza11**	**Sids12**
Control	0	52.8 ^g^	40.9 ^o^	30.6 ^y^	54.5 ^i^	43.1 ^q^	31.9 ^y^
Salicylic acid	250	62.2 ^b^	49.1 ^i^	37.7 ^r^	62.9 ^c^	51.3 ^k^	39.1 ^t^
	500	64.4 ^a^	51.3 ^h^	39.3 ^q^	64.4 ^b^	53.0 ^j^	40.5 ^s^
	1000	64.6 ^a^	51.4 ^h^	39.8 ^p^	65.3 ^a^	53.3 ^j^	41.1 ^r^
Indole acetic acid	25	58.6 ^d^	45.6 ^l^	34.7 ^u^	59.3 ^e^	48.2 ^n^	36.0 ^v^
	50	60.8 ^c^	47.4 ^k^	36.2 ^t^	61.4 ^d^	49.6 ^m^	37.7 ^u^
	100	61.0 ^c^	47.9 ^j^	36.7 ^s^	61.7 ^d^	50.3 ^l^	38.1 ^u^
Oxalic acid	250	55.0 ^f^	42.1 ^n^	31.6 ^x^	55.7 ^h^	45.2 ^p^	32.9 ^x^
	500	57.2 ^e^	44.2 ^m^	33.3 ^w^	57.5 ^g^	46.8 ^o^	34.6 ^w^
	1000	57.4 ^e^	44.4 ^m^	33.7 ^v^	58.1 ^f^	47.2 ^o^	35.0 ^w^
L.S.D.							
Varieties		0.231			0.097		
Inducers		0.172			0.284		
Varieties × Inducers		0.361			0.476		

The letters assigned (e.g., ‘a’, ‘b’, ‘c’) correspond to the results of Duncan’s multiple range test conducted at a significance level of *p* < 0.05. Similar letters denote non-significance, while dissimilar letters denote significant differences between treatments. × refers to the interaction between variables.

**Table 5:** (**a**) GR enzyme activity on wheat plants infected by stripe rust before (pre-) and after (post-) inoculation with some fungicide (Tilit 25% EC, Montoro 30% EC, and Sumi eight 5% EC) for three wheat varieties (Misr1, Gimmeiza11, and Sids12). (**b**) Comparison of GR activity in three wheat varieties (Misr1, Gimmeiza11, and Sids12) infected with stripe rust and treated with chemical inducers (salicylic acid, indole acetic acid, and oxalic acid) pre- and post-inoculation.

**(a)**							
**Fungicides**	**Concentration (ppm)**	**Pre-Inoculation**			**Post-Inoculation**		
		**Misr1**	**Gimmeiza11**	**Sids12**	**Misr1**	**Gimmeiza11**	**Sids12**
Control	0	51.4 ^j^	38.6 ^r^	26.4 ^z^	53.4 ^g^	40.5 ^p^	28.4 ^y^
Tilit	125	61.7 ^c^	47.8 ^l^	34.9 ^u^	62.8 ^b^	49.7 ^j^	37 ^r^
	250	64.2 ^b^	50.1 ^k^	36.8 ^t^	65.1 ^a^	51.7 ^i^	39.2 ^q^
	500	64.6 ^a^	50.4 ^k^	37.3 ^s^	65.5 ^a^	52.3 ^h^	39.4 ^q^
Montoro	125	57.3 ^f^	44 ^n^	31.3 ^w^	58.9 ^d^	45.9 ^l^	33.4 ^u^
	250	59.1 ^e^	46.3 ^m^	33.5 ^v^	61.2 ^c^	48.2 ^k^	34.9 ^t^
	500	60.2 ^C^	46.5 ^m^	33.7 ^v^	61.5 ^c^	48.4 ^k^	35.8 ^s^
Sumi 8	125	52.9 ^i^	40.1 ^q^	27.7 ^y^	54.9 ^f^	42 ^o^	29.8 ^x^
	250	55.4 ^h^	42 ^p^	29.9 ^x^	57.2 ^e^	43.9 ^n^	31.7 ^w^
	500	55.8 ^g^	42.7 ^o^	30.1 ^x^	57.5 ^e^	44.6 ^m^	32.2 ^v^
L.S.D.							
Varieties		0.048			0.179		
Fungicides		0.173			0.192		
Varieties × Fungicides		0.289			0.361		
**(b)**							
**Chemical Inducers**	**Concentration (ppm)**	**Pre-Inoculation**			**Post-Inoculation**		
		**Misr1**	**Gimmeiza11**	**Sids12**	**Misr1**	**Gimmeiza11**	**Sids12**
Control	0	51.0 ^g^	37.5 ^n^	25.5 ^w^	53.0 ^h^	40.1 ^o^	27.2 ^x^
Salicylic acid	250	61.3 ^b^	46.9 ^i^	33.9 ^q^	62.1 ^b^	49.2 ^j^	35.9 ^r^
	500	64.1 ^a^	49.5 ^h^	35.9 ^p^	64.4 ^a^	51.5 ^i^	37.1 ^q^
	1000	64.3 ^a^	49.6 ^h^	36.3 ^o^	64.6 ^a^	51.7 ^i^	38.0 ^p^
Indole acetic acid	25	58.2 ^d^	44.1 ^k^	30.3 ^s^	58.2 ^d^	45.3 ^l^	32.4 ^t^
	50	58.4 ^d^	44.2 ^k^	32.5 ^r^	60.5 ^c^	45.5 ^l^	34.6 ^s^
	100	59.9 ^c^	45.6 ^j^	32.7 ^r^	60.8 ^c^	47.9 ^k^	35.0 ^s^
Oxalic acid	250	52.5 ^f^	38.5 ^m^	27.0 ^v^	54.3 ^g^	41.1 ^n^	28.8 ^w^
	500	55.2 ^e^	41.4 ^l^	27.9 ^u^	56.3 ^f^	43.7 ^m^	29.3 ^v^
	1000	55.4 ^e^	41.5 ^l^	29.1 ^t^	56.9 ^e^	44 ^m^	31.1 ^u^
L.S.D.							
Varieties		0.112			0.108		
Inducers		0.194			0.188		
Varieties × Inducers		0.337			0.376		

The letters assigned (e.g., ‘a’, ‘b’, ‘c’) correspond to the results of Duncan’s multiple range test conducted at a significance level of *p* < 0.05. Dissimilar letters signify significant differences between treatments, while identical letters denote non-significance. × refers to the interaction between variables.

**Table 6:** (**a**) SOD enzyme activity on wheat plants infected by stripe rust before (pre-) and after (post-) inoculation with some fungicide (Tilit 25% EC, Montoro 30% EC, and Sumi eight 5% EC) for three wheat varieties (Misr1, Gimmeiza11, and Sids12). (**b**) Comparative analysis of SOD activity in three wheat varieties (Misr1, Gimmeiza11, and Sids12) inoculated with stripe rust and treated with chemical inducers (salicylic acid, indole acetic acid, and oxalic acid.

**(a)**							
**Fungicides**	**Concentration (ppm)**	**Pre-Inoculation**			**Post-Inoculation**		
		**Misr1**	**Gimmeiza11**	**Sids12**	**Misr1**	**Gimmeiza11**	**Sids12**
Control	0	8.38 ^h^	6.31 ^o^	4.20 ^v^	8.57 ^h^	6.51 ^q^	4.40 ^z^
Tilit	125	9.56 ^b^	7.99 ^j^	5.84 ^q^	9.76 ^b^	8.10 ^k^	6.01 ^t^
	250	9.59 ^b^	8.00 ^j^	5.89 ^q^	9.78 ^b^	8.19 ^j^	6.09 ^s^
	500	9.74 ^a^	8.21 ^i^	6.10 ^p^	9.93 ^a^	8.40 ^i^	6.30 ^r^
Montoro	125	8.98 ^e^	7.35 ^l^	5.21 ^s^	9.24 ^e^	7.51 ^m^	5.38 ^w^
	250	9.13 ^k^	7.39 ^l^	5.26 ^s^	9.32 ^d^	7.56 ^m^	5.46 ^v^
	500	9.28 ^c^	7.58 ^k^	5.47 ^r^	9.47 ^c^	7.77 ^l^	5.67 ^u^
Sumi 8	125	8.68 ^g^	6.68 ^n^	4.54 ^u^	8.85 ^g^	6.85 ^p^	4.77 ^y^
	250	8.69 ^g^	6.73 ^n^	4.59 ^u^	8.87 ^g^	6.93 ^o^	4.82 ^y^
	500	8.83 ^f^	6.94 ^m^	4.83 ^t^	9.03 ^f^	7.16 ^n^	5.03 ^x^
L.S.D.							
Varieties		0.022			0.031		
Fungicides		0.029			0.045		
Varieties × Fungicides		0.053			0.067		
**(b)**							
**Chemical Inducers**	**Concentration (ppm)**	**Pre-Inoculation**			**Post-Inoculation**		
		**Misr1**	**Gimmeiza11**	**Sids12**	**Misr1**	**Gimmeiza11**	**Sids12**
Control	0	7.56 ^h^	6.00 ^p^	3.99 ^y^	7.93 ^h^	6.14 ^q^	4.35 ^z^
Salicylic acid	250	8.64 ^b^	7.24 ^j^	5.46 ^s^	8.98 ^b^	7.40 ^j^	5.60 ^s^
	500	8.94 ^a^	7.26 ^j^	5.84 ^r^	9.26 ^a^	7.74 ^i^	5.92 ^r^
	1000	8.95 ^a^	7.41 ^i^	5.89 ^q^	9.29 ^a^	7.76 ^i^	5.95 ^r^
Indole acetic acid	25	8.21 ^e^	6.77 ^m^	4.83 ^u^	8.53 ^e^	6.86 ^m^	5.07 ^v^
	50	8.42 ^d^	6.92 ^l^	5.24 ^t^	8.78 ^d^	7.15 ^l^	5.35 ^u^
	100	8.49 ^c^	7.00 ^k^	5.28 ^t^	8.83 ^c^	7.22 ^k^	5.43 ^t^
Oxalic acid	250	7.81 ^g^	6.25 ^o^	4.24 ^x^	8.08 ^g^	6.32 ^p^	4.53 ^y^
	500	7.94 ^f^	6.48 ^n^	4.34 ^w^	8.36 ^f^	6.63 ^o^	4.81 ^x^
	1000	7.98 ^f^	6.51 ^n^	4.62 ^v^	8.38 ^f^	6.68 ^n^	4.89 ^w^
LSD.							
Varieties		0.025			0.021		
Inducers		0.029			0.024		
Varieties × Inducers		0.051			0.042		

The letters assigned (e.g., ‘a’, ‘b’, ‘c’) correspond to the results of Duncan’s multiple range test conducted at a significance level of *p* < 0.05. The different letters denoting significant differences between treatments, while similar letters denote nonsignificance. × refers to the interaction between variables.

**Table 7:** (**a**) POX enzyme activity on wheat plants infected by stripe rust before (pre-) and after (post-) inoculation with some fungicide (Tilit 25% EC, Montoro 30% EC, and Sumi eight 5% EC) for three wheat varieties (Misr1, Gimmeiza11, and Sids12). (**b**) Comparison of POX activity response in three wheat varieties (Misr1, Gimmeiza11, and Sids12) to chemical inducers (salicylic acid, indole acetic acid, and oxalic acid) under stripe rust infection.

**(a)**							
**Fungicides**	**Concentration (ppm)**	**Pre-Inoculation**			**Post-Inoculation**		
		**Misr1**	**Gimmeiza11**	**Sids12**	**Misr1**	**Gimmeiza11**	**Sids12**
Control	0	4.97 ^i^	3.17 ^p^	1.93 ^x^	5.27 ^i^	3.47 ^r^	2.23 ^y^
Tilit	125	6.23 ^c^	4.43 ^k^	2.80 ^s^	6.53 ^c^	4.73 ^l^	3.07 ^t^
	250	6.51 ^b^	4.74 ^j^	2.92 ^r^	6.81 ^b^	4.96 ^k^	3.29 ^s^
	500	6.59 ^a^	4.79 ^j^	3.01 ^q^	6.89 ^a^	5.09 ^j^	3.31 ^s^
Montoro	125	5.69 ^f^	3.89 ^m^	2.41 ^u^	5.99 ^f^	4.19 ^o^	2.71 ^v^
	250	5.94 ^e^	4.20 ^l^	2.63 ^t^	6.27 ^e^	4.43 ^n^	2.93 ^u^
	500	6.05 ^d^	4.25 ^l^	2.65 ^t^	6.35 ^d^	4.55 ^m^	2.95 ^u^
Sumi 8	125	5.15 ^h^	3.35 ^o^	2.05 ^w^	5.45 ^h^	3.65 ^q^	2.35 ^x^
	250	5.49 ^g^	3.69 ^n^	2.23 ^v^	5.79 ^g^	3.96 ^p^	2.53 ^w^
	500	5.49 ^g^	3.71 ^n^	2.29 ^v^	5.81 ^g^	4.01 ^p^	2.59 ^w^
L.S.D.							
Varieties		0.023			0.037		
Fungicides		0.032			0.035		
Varieties × Fungicides		0.058			0.068		
**(b)**							
**Chemical Inducers**	**Concentration (ppm)**	**Pre-Inoculation**			**Post-Inoculation**		
		**Misr1**	**Gimmeiza11**	**Sids12**	**Misr1**	**Gimmeiza11**	**Sids12**
Control	0	4.49 ^h^	3.02 ^q^	1.49 ^x^	4.87 ^h^	3.37 ^q^	1.87 ^x^
Salicylic acid	250	5.54 ^b^	4.10 ^k^	2.54 ^s^	5.92 ^c^	4.42 ^j^	2.92 ^s^
	500	5.79 ^a^	4.25 ^j^	2.79 ^r^	6.16 ^b^	4.67 ^i^	3.16 ^r^
	1000	5.84 ^a^	4.34 ^i^	2.84 ^r^	6.22 ^a^	4.72 ^i^	3.22 ^r^
Indole acetic acid	25	5.12 ^d^	3.59 ^m^	2.12 ^u^	5.47 ^e^	4.06 ^m^	2.47 ^u^
	50	5.37 ^c^	3.84 ^l^	2.17 ^u^	5.75 ^d^	4.14 ^l^	2.72 ^t^
	100	5.39 ^c^	3.89 ^l^	2.39 ^t^	5.77 ^d^	4.27 ^k^	2.77 ^t^
Oxalic acid	250	4.67 ^g^	3.14 ^p^	1.64 ^w^	5.08 ^g^	3.52 ^p^	2.08 ^w^
	500	4.85 ^f^	3.33 ^o^	1.65 ^w^	5.12 ^g^	3.73 ^o^	2.27 ^v^
	1000	4.94 ^e^	3.44 ^n^	1.94 ^v^	5.32 ^f^	3.82 ^n^	2.32 ^v^
L.S.D.							
Varieties		0.027			0.028		
Inducers		0.031			0.033		
varieties × Inducers		0.054			0.057		

The letters assigned in accordance with Duncan’s analysis at a significance level of *p* < 0.05 serve to highlight statistically significant differences among treatments, whereas shared letters denote non-statistically significant. × refers to the interaction between variables.

**Table 8:** Three applied fungicides utilized to control wheat stripe rust.

SN	Common Name	Trade Name	Active Ingredient
1	Propiconazole	Tilt	25 EC
2	Difenoconazole + Propiconazole	Montoro	30 EC
3	Diniconazole	Sumi eight	5 EC

**Table 9:** Seedling infection types of wheat stripe rust [104].

Infection Type	Infection Class	Symptoms
0	Immune	No visible symptoms
1	High resistant	Necrotic flecks without sporulation
2	Resistant	Necrosis without sporulation
3	Moderately resistant	Necrosis with trace sporulation
4	Light–moderate	Light sporulation surrounded by necrosis
5	Moderate	Intermediate sporulation with necrosis/chlorosis
6	High–moderate	Moderate sporulation surrounded by chlorosis
7	Moderately susceptible	Moderate sporulation with moderate chlorosis
8	Susceptible	Sufficient sporulation with little or no chlorosis
9	Very susceptible	Abundant sporulation without chlorosis

## Data Availability

The data substantiating our results has been made publicly available and can be accessed through the following link: https://doi.org/10.6084/m9.figshare.23896944.

## References

[1] Beddow J.M., Pardey P.G., Chai Y., Hurley T.M., Kriticos D.J., Braun H.-J., Park R.F., Cuddy W.S., Yonow T. (2015). Research investment implications of shifts in the global geography of wheat stripe rust. Nat. Plants.

[2] Figueroa M., Hammond-Kosack K.E., Solomon P.S. (2018). A review of wheat diseases-a field perspective. Mol. Plant Pathol..

[3] Chen W., Wellings C., Chen X., Kang Z., Liu T. (2014). Wheat stripe (yellow) rust caused by *Puccinia striiformis* f. sp. *tritici*. Mol. Plant Pathol..

[4] Thirugnana Sambandham V., Shankar P., Mukhopadhaya S. (2022). Early Onset Yellow Rust Detection Guided by Remote Sensing Indices. Agriculture.

[5] Omara R.I., Mazrou Y.S., Elsayed A., Moawad N., Nehela Y., Shahin A.A. (2022). MISSR: A Mentoring Interactive System for Stripe Rust. Agronomy.

[6] Shahin A., Ashmawy M., El-Orabey W., Esmail S. (2020). Yield losses in wheat caused by stripe rust (*Puccinia striiformis*) in Egypt. Am. J. Life Sci..

[7] Mishra A., Tiwari K., Prakasha T., Prasad S.S. (2021). Use of Host Resistance for Management Wheat Rusts. Innovative Approaches in Diagnosis and Management of Crop Diseases.

[8] Shahin A.A. (2020). Occurrence of new races and virulence changes of the wheat stripe rust pathogen (*Puccinia striiformis* f. sp. *tritici*) in Egypt. Arch. Phytopathol. Plant Prot..

[9] Jørgensen L.N., Hovmøller M.S., Hansen J.G., Lassen P., Clark B., Bayles R., Rodemann B., Flath K., Jahn M., Goral T. (2014). IPM strategies and their dilemmas including an introduction to www.eurowheat.org. J. Integr. Agric..

[10] Viljanen-Rollinson S., Marroni M., Butler R. (2006). Wheat stripe rust control using fungicides in New Zealand. N. Z. Plant Prot..

[11] Khan M.A., Raheel M., Khan S.A., Abid A.D., Shahzad S., Siddiqui H.Z., Atif M., Hanif A. (2023). Eco-friendly management of wheat stripe rust through application of *Bacillus subtilis* in combination with plant defense activators. J. King Saud Univ.-Sci..

[12] Chen X., Kang Z. (2017). Integrated Control of Stripe Rust.

[13] Zeng Q., Zhao J., Wu J., Zhan G., Han D., Kang Z. (2022). Wheat stripe rust and integration of sustainable control strategies in China. Front. Agric. Sci. Eng..

[14] Kiani T., Mehboob F., Hyder M.Z., Zainy Z., Xu L., Huang L., Farrakh S. (2021). Control of stripe rust of wheat using indigenous endophytic bacteria at seedling and adult plant stage. Sci. Rep..

[15] Bale J., Van Lenteren J., Bigler F. (2008). Biological control and sustainable food production. Philos. Trans. R. Soc. B Biol. Sci..

[16] Hermans S.M., Buckley H.L., Case B.S., Curran-Cournane F., Taylor M., Lear G. (2020). Using soil bacterial communities to predict physico-chemical variables and soil quality. Microbiome.

[17] Archana H., Darshan K., Lakshmi M.A., Ghoshal T., Bashayal B.M., Aggarwal R. (2022). Biopesticides: A key player in agro-environmental sustainability. Trends of Applied Microbiology for Sustainable Economy.

[18] Aboulila A.A. (2022). Efficiency of plant growth regulators as inducers for improve systemic acquired resistance against stripe rust disease caused by *Puccinia striiformis* f. sp. *tritici* in wheat through up-regulation of PR-1 and PR-4 genes expression. Physiol. Mol. Plant Pathol..

[19] Heil M., Bostock R.M. (2002). Induced systemic resistance (ISR) against pathogens in the context of induced plant defences. Ann. Bot..

[20] Hembade V.L., Yashveer S., Taunk J., Sangwan S., Tokas J., Singh V., Redhu N.S., Grewal S., Malhotra S., Kumar M. (2022). Chitosan-Salicylic acid and Zinc sulphate nano-formulations defend against yellow rust in wheat by activating pathogenesis-related genes and enzymes. Plant Physiol. Biochem..

[21] Alexandersson E., Mulugeta T., Lankinen Å., Liljeroth E., Andreasson E. (2016). Plant resistance inducers against pathogens in Solanaceae species-from molecular mechanisms to field application. Int. J. Mol. Sci..

[22] Chen X. (2020). Pathogens which threaten food security: *Puccinia striiformis*, the wheat stripe rust pathogen. Food Secur..

[23] Li H., Zhao J., Feng H., Huang L., Kang Z. (2013). Biological control of wheat stripe rust by an endophytic *Bacillus subtilis* strain E1R-j in greenhouse and field trials. Crop Prot..

[24] Pang F., Wang T., Zhao C., Tao A., Yu Z., Huang S., Yu G. (2016). Novel bacterial endophytes isolated from winter wheat plants as biocontrol agent against stripe rust of wheat. BioControl.

[25] Reiss A., Jørgensen L.N. (2017). Biological control of yellow rust of wheat (*Puccinia striiformis*) with Serenade^®^ ASO (*Bacillus subtilis* strain QST713). Crop Prot..

[26] Omara R.I., Essa T.A., Khalil A.A., Elsharkawy M.M. (2020). A case study of non-traditional treatments for the control of wheat stem rust disease. Egypt. J. Biol. Pest Control.

[27] Dannies M., Tugizimana F., Steenkamp P.A., Piater L.A., Dubery I.A., Terefe T., Mhlongo M.I. (2023). Metabolomic evaluation of PGPR defence priming in wheat (*Triticum aestivum* L.) cultivars infected with *Puccinia striiformis* f. sp. *tritici* (stripe rust). Front. Plant Sci..

[28] Loeffler W., Tschen J.S.M., Vanittanakom N., Kugler M., Knorpp E., Hsieh T.F., Wu T.G. (1986). Antifungal effects of bacilysin and fengymycin from *Bacillus subtilis* F-29-3 a comparison with activities of other *Bacillus* antibiotics. J. Phytopathol..

[29] Wang C., Zhang J., Chen H., Fan Y., Shi Z. (2010). Antifungal activity of eugenol against *Botrytis cinerea*. Trop. Plant Pathol..

[30] Abo-Elyousr K.A., Abdel-Rahim I.R., Almasoudi N.M., Alghamdi S.A. (2021). Native endophytic Pseudomonas putida as a biocontrol agent against common bean rust caused by Uromyces appendiculatus. J. Fungi.

[31] Abebe W. (2021). Wheat leaf rust disease management: A Review. Int. J. Novel Res. Interdiscip. Stud..

[32] Tjamos S.E., Flemetakis E., Paplomatas E.J., Katinakis P. (2005). Induction of resistance to Verticillium dahliae in Arabidopsis thaliana by the biocontrol agent K-165 and pathogenesis-related proteins gene expression. Mol. Plant-Microbe Interact..

[33] Shatrupa Ray S.R., Vivek Singh V.S., Kartikay Bisen K.B., Chetan Keswani C.K., Surendra Singh S.S., Singh H. (2017). Endophytomicrobiont: A multifaceted beneficial interaction. Advances in PGPR Research.

[34] Sivasakthi S., Usharani G., Saranraj P. (2014). Biocontrol potentiality of plant growth promoting bacteria (PGPR)-Pseudomonas fluorescens and *Bacillus subtilis*: A review. Afr. J. Agric. Res..

[35] Zehra A., Raytekar N.A., Meena M., Swapnil P. (2021). Efficiency of microbial bio-agents as elicitors in plant defense mechanism under biotic stress: A review. Curr. Res. Microb. Sci..

[36] Urszula W., Barbara M., Monika B., Zofia K. (2004). Biological control of winter wheat pathogens by bacteria. Acta Fytotech. Zootech..

[37] El-Kazzaz M.K., Ghoniem K.E., Ashmawy M.A., Omar G.E., Hafez Y.M. (2020). Suppression of wheat strip rust disease caused by *Puccinia striiformis* f. sp. *tritici* by eco-friendly bio-control agents correlated with yield improvement. Fresenius Environ. Bull..

[38] Hafez Y., Emeran A., Esmail S., Mazrou Y., Abdrabbo D., Abdelaal K. (2020). Alternative treatments improve physiological characters, yield and tolerance of wheat plants under leaf rust disease stress. Fresenius Environ. Bull..

[39] Esmail S.M., Draz I.S., Saleem M.H., Mumtaz S., Elsharkawy M.M. (2022). *Penicillium simplicissimum* and *Trichoderma asperellum* counteract the challenge of *Puccinia striiformis* f. sp. *tritici* in wheat plants. Egypt. J. Biol. Pest Control.

[40] Singh V.K., Mathuria R., Gogoi R., Aggarwal R. (2016). Impact of different fungicides and bioagents, and fungicidal spray timing on wheat stripe rust development and grain yield. Indian Phytopathol..

[41] Abu El-Naga S., Khalifa M., Sherif S., Youssef W., El-Daoudi Y., Shafik I. Virulence of wheat stripe rust pathotypes identified in Egypt during 1999/2000 and sources of resistance. Proceedings of the Meeting the Challenge of Yellow Rust in Cereal Crops, Proceedings of the First Regional Conference on Yellow Rust in the Central and West Asia and North Africa Region.

[42] Omara R., El-Naggar D., El-Malik A., Ketta H. (2016). Losses assessment in some Egyptian wheat cultivars caused by stripe rust pathogen (*Puccinia striiformis*). Egypt. J. Phytopathol..

[43] Esmail S.M., Omara R.I., Abdelaal K.A., Hafez Y.M. (2019). Histological and biochemical aspects of compatible and incompatible wheat-*Puccinia striiformis* interactions. Physiol. Mol. Plant Pathol..

[44] Abdelaal K.A., Hafez Y., Badr M., Youseef W., Esmail S.M. (2014). Biochemical, Histological and Molecular Changes in Susceptible and Resistant Wheat Cultivars Inoculated with Stripe Rust Fungus *Puccinia striiformis* f. sp. *tritici*. Egypt. J. Biol. Pest Control.

[45] Singh R., Huerta-Espino J., Rajaram S. (2000). Achieving near-immunity to leaf and stripe rusts in wheat by combining slow rusting resistance genes. Acta Phytopathol. Entomol. Hung..

[46] Zheng S., Li Y., Lu L., Liu Z., Zhang C., Ao D., Li L., Zhang C., Liu R., Luo C. (2017). Evaluating the contribution of Yr genes to stripe rust resistance breeding through marker-assisted detection in wheat. Euphytica.

[47] Abu Aly A., Omara R., El-Malik A., Nagwa I. (2017). Evaluation of new sources of resistance to wheat stripe rust (*Puccinia striiformis* f. sp. *tritici*), under Egyptian field conditions. J. Plant Prot. Pathol..

[48] Coram T.E., Wang M., Chen X. (2008). Transcriptome analysis of the wheat-*Puccinia striiformis* f. sp. *tritici* interaction. Mol. Plant Pathol..

[49] Liu R., Lu J., Zhou M., Zheng S., Liu Z., Zhang C., Du M., Wang M., Li Y., Wu Y. (2020). Developing stripe rust resistant wheat (*Triticum aestivum* L.) lines with gene pyramiding strategy and marker-assisted selection. Genet. Resour. Crop Evol..

[50] Khan N., Ali S., Shahid M.A., Kharabian-Masouleh A. (2017). Advances in detection of stress tolerance in plants through metabolomics approaches. Plant Omics.

[51] Cheng Y., Zhang H., Yao J., Han Q., Wang X., Huang L., Kang Z. (2013). Cytological and molecular characterization of non-host resistance in Arabidopsis thaliana against wheat stripe rust. Plant Physiol. Biochem..

[52] Bano A. (2020). Interactive effects of Ag-nanoparticles, salicylic acid, and plant growth promoting rhizobacteria on the physiology of wheat infected with yellow rust. J. Plant Pathol..

[53] Elsharkawy M.M., Omara R.I., Mostafa Y.S., Alamri S.A., Hashem M., Alrumman S.A., Ahmad A.A. (2022). Mechanism of wheat leaf rust control using chitosan nanoparticles and salicylic acid. J. Fungi.

[54] Fayadh A.H., Al-Maaroof E.M., Fattah F.A. (2013). Induced resistance to wheat yellow rust by chemical inducers. J. Biol. Agric. Healthc..

[55] Corredor-Moreno P., Minter F., Davey P.E., Wegel E., Kular B., Brett P., Lewis C.M., Morgan Y.M., Macías Pérez L.A., Korolev A.V. (2021). The branched-chain amino acid aminotransferase TaBCAT1 modulates amino acid metabolism and positively regulates wheat rust susceptibility. Plant Cell.

[56] Khalil M.S.A., Abdel-Kader M.M., El-Mougy N.S., El-Gamal N.G. (2021). Foliar application with organic acids for suppressing the severity of wheat powdery mildew disease caused by Blumeria graminis f. sp. *tritici* under field conditions. Res. Crops.

[57] Devi B., Singh G., Dash A.K., Gupta S. (2020). Chemically induced systemic acquired resistance in the inhibition of French bean rust. Curr. Plant Biol..

[58] Han Q.-M., Kang Z.-S., Wei G.-R. (2003). Studies on Fulicur and Caramba for controlling wheat stripe rust disease. Plant Prot.-Beijing.

[59] Chen X. (2005). Epidemiology and control of stripe rust [*Puccinia striiformis* f. sp. *tritici*] on wheat. Can. J. Plant Pathol..

[60] Wan A.M., Chen X.M., He Z. (2007). Wheat stripe rust in China. Aust. J. Agric. Res..

[61] Han Q., Kang Z., Buchenauer H., Huang L., Zhao J. (2006). Cytological and immunocytochemical studies on the effects of the fungicide tebuconazole on the interaction of wheat with stripe rust. J. Plant Pathol..

[62] Hamada M.S., Yin Y., Ma Z. (2011). Sensitivity to iprodione, difenoconazole and fludioxonil of Rhizoctonia cerealis isolates collected from wheat in China. Crop Prot..

[63] Wang F., Cao D., Shi L., He S., Li X., Fang H., Yu Y. (2020). Competitive adsorption and mobility of propiconazole and difenoconazole on five different soils. Bull. Environ. Contam. Toxicol..

[64] Carmona M., Sautua F., Pérez-Hérnandez O., Reis E.M. (2020). Role of fungicide applications on the integrated management of wheat stripe rust. Front. Plant Sci..

[65] Cook N.M., Chng S., Woodman T.L., Warren R., Oliver R.P., Saunders D.G. (2021). High frequency of fungicide resistance-associated mutations in the wheat yellow rust pathogen *Puccinia striiformis* f. sp. *tritici*. Pest Manag. Sci..

[66] Steffens J.J., Pell E.J., Tien M. (1996). Mechanisms of fungicide resistance in phytopathogenic fungi. Curr. Opin. Biotechnol..

[67] Jørgensen L.N., Matzen N., Hansen J.G., Semaskiene R., Korbas M., Danielewicz J., Glazek M., Maumene C., Rodemann B., Weigand S. (2018). Four azoles’ profile in the control of Septoria, yellow rust and brown rust in wheat across Europe. Crop Prot..

[68] Godeau C., Morin-Crini N., Staelens J.-N., Martel B., Rocchi S., Chanet G., Fourmentin M., Crini G. (2021). Adsorption of a triazole antifungal agent, difenoconazole, on soils from a cereal farm: Protective effect of hemp felt. Environ. Technol. Innov..

[69] Zhang Z., Jiang W., Jian Q., Song W., Zheng Z., Wang D., Liu X. (2015). Residues and dissipation kinetics of triazole fungicides difenoconazole and propiconazole in wheat and soil in Chinese fields. Food Chem..

[70] Chen X. (2014). Integration of cultivar resistance and fungicide application for control of wheat stripe rust. Can. J. Plant Pathol..

[71] Chen X., Evans C., Garner J., Liu Y. (2013). Control of stripe rust of spring wheat with foliar fungicide. Plant Disease.

[72] Ali Y., Abbas T., Aatif H.M., Ahmad S., Khan A.A., Hanif C.M. (2022). Impact of foliar applications of different fungicides on wheat stripe rust epidemics and grain yield. Pak. J. Phytopathol..

[73] Bekana N.B. (2019). Efficacy evaluation of different foliar fungicides for the management of wheat strip rust (*Puccinia striiformis*) in West Shoa Zone, Oromia, Ethiopia. J. Appl. Sci. Environ. Manag..

[74] Jindal M.M., Sharma I., Bains N.S. (2012). Losses due to stripe rust caused by *Puccinia striiformis* in different varieties of wheat. J. Cereal Res..

[75] Basandrai A.K., Sharma B., Basandrai D. (2013). Efficacy of triazole fungicides for the integrated management of yellow rust, leaf rust and powdery mildew of wheat. Plant Dis. Res..

[76] Kang Z., Li X., Wan A., Wang M., Chen X. (2019). Differential sensitivity among *Puccinia striiformis* f. sp. *tritici* isolates to propiconazole and pyraclostrobin fungicides. Can. J. Plant Pathol..

[77] Basandrai A.K., Mehta A., Rathee V., Basandrai D., Sharma B. (2020). Efficacy of fungicides in managing yellow rust of wheat. J. Cereal Res..

[78] Covarelli L., Orfei M. (2005). Chemical control of foliar disease of winter bread wheat. Inf. Fitopatlolgico.

[79] Walters D.R., Avrova A., Bingham I.J., Burnett F.J., Fountaine J., Havis N.D., Hoad S.P., Hughes G., Looseley M., Oxley S.J. (2012). Control of foliar diseases in barley: Towards an integrated approach. Eur. J. Plant Pathol..

[80] Rodríguez-García M.F., González-González M., Huerta-Espino J., Solano-Hernández S. (2021). Fungicides evaluation against yellow rust (*Puccinia striiformis* f. sp. hordei) in six barley cultivars. Rev. Mex. Fitopatol..

[81] Boualem B., Mohamed B., Moulay B. (2017). Effect of application timing of artea and amistar xtra on the yield of wheat (*Triticum aestivum* L.) under foliar disease in the East-Algerian. Int. J. Agric. Res..

[82] Omara R., Kamel S., Hafez Y., Morsy S. (2015). Role of Non-traditional Control Treatments in Inducing Resistance Against Wheat Leaf Rust Caused by *Puccinia triticina*. Egypt. J. Biol. Pest Control.

[83] Hanifei M., Dehghani H., Choukan R. (2013). The role of antioxidant enzymes and phenolic compounds in disease resistance to *Fusarium oxysporum* f. sp. Melonis race 1.2. Int. J. Agron. Plant Prod..

[84] Rizhsky L., Hallak-Herr E., Van Breusegem F., Rachmilevitch S., Barr J.E., Rodermel S., Inzé D., Mittler R. (2002). Double antisense plants lacking ascorbate peroxidase and catalase are less sensitive to oxidative stress than single antisense plants lacking ascorbate peroxidase or catalase. Plant J..

[85] Liau C.Y., Lin C.S. (2008). Detection of chitinolytic enzymes in Ipomoea batatas leaf extract by activity staining after gel electrophoresis. J. Chin. Chem. Soc..

[86] Khalifa N.A., Abou-Zeid N., Noher A.M., Abbas M., Sobhy H. (2016). Enzyme activity and biochemical changes associated with induction of systemic resistance of faba bean against damping off disease. Egypt. J. Biol. Pest Control.

[87] Trchounian A., Petrosyan M., Sahakyan N. (2016). Plant cell redox homeostasis and reactive oxygen species. Redox State as a Central Regulator of Plant-Cell Stress Responses.

[88] Milavec M., Ravnikar M., Kovač M. (2001). Peroxidases and photosynthetic pigments in susceptible potato infected with potato virus YNTN. Plant Physiol. Biochem..

[89] Hameed F.E., Abood J.K., Temur H.A. (2011). The change of peroxidase activity in three cucumber cultivars during development of powdery mildew infection. J. Babylon. Univ. Pure Appl. Sci..

[90] El-Komy M.H. (2014). Comparative analysis of defense responses in chocolate spot-resistant and-susceptible faba bean (*Vicia faba*) cultivars following infection by the necrotrophic fungus Botrytis fabae. Plant Pathol. J..

[91] Zhang Z., Yang D., Yang B., Gao Z., Li M., Jiang Y., Hu M. (2013). β-Aminobutyric acid induces resistance of mango fruit to postharvest anthracnose caused by Colletotrichum gloeosporioides and enhances activity of fruit defense mechanisms. Sci. Hortic..

[92] Foyer C.H., Noctor G. (2013). Redox Signaling in Plants. Antioxid Redox Signal..

[93] Martinez V., Mestre T.C., Rubio F., Girones-Vilaplana A., Moreno D.A., Mittler R., Rivero R.M. (2016). Accumulation of flavonols over hydroxycinnamic acids favors oxidative damage protection under abiotic stress. Front. Plant Sci..

[94] Abdelaal K.A. (2015). Effect of salicylic acid and abscisic acid on morpho-physiological and anatomical characters of faba bean plants (*Vicia faba* L.) under drought stress. J. Plant Prod..

[95] Gupta G., Parihar S.S., Ahirwar N.K., Snehi S.K., Singh V. (2015). Plant growth promoting rhizobacteria (PGPR): Current and future prospects for development of sustainable agriculture. J. Microb. Biochem. Technol..

[96] Liu R., Li J., Zhang L., Feng T., Zhang Z., Zhang B. (2021). Fungicide difenoconazole induced biochemical and developmental toxicity in wheat (*Triticum aestivum* L.). Plants.

[97] Apel K., Hirt H. (2004). Reactive oxygen species: Metabolism, oxidative stress, and signal transduction. Annu. Rev. Plant Biol..

[98] Khaledi N., Taheri P., Falahati-Rastegar M. (2017). Evaluation of resistance and the role of some defense responses in wheat cultivars to Fusarium head blight. J. Plant Prot. Res..

[99] Feng H., Wang X., Zhang Q., Fu Y., Feng C., Wang B., Huang L., Kang Z. (2014). Monodehydroascorbate reductase gene, regulated by the wheat PN-2013 miRNA, contributes to adult wheat plant resistance to stripe rust through ROS metabolism. Biochim. Biophys. Acta (BBA)-Gene Regul. Mech..

[100] Lagudah E.S., Krattinger S.G., Herrera-Foessel S., Singh R.P., Huerta-Espino J., Spielmeyer W., Brown-Guedira G., Selter L.L., Keller B. (2009). Gene-specific markers for the wheat gene Lr34/Yr18/Pm38 which confers resistance to multiple fungal pathogens. Theor. Appl. Genet..

[101] Elsharkawy M.M., Shimizu M., Takahashi H., Ozaki K., Hyakumachi M. (2013). Induction of systemic resistance against Cucumber mosaic virus in Arabidopsis thaliana by *Trichoderma asperellum* SKT-1. Plant Pathol. J..

[102] Levy A., Guenoune-Gelbart D., Epel B.L. (2007). β-1,3-Glucanases: Plasmodesmal gate keepers for intercellular communication. Plant Signal. Behav..

[103] Wan A., Zhao Z., Chen X., He Z., Jin S., Jia Q., Yao G., Yang J., Wang B., Li G. (2004). Wheat stripe rust epidemic and virulence of *Puccinia striiformis* f. sp. *tritici* in China in 2002. Plant Dis..

[104] McNeal F., Konzak C., Smith E., Tate W., Russell T. (1971). A Uniform System for Recording and Processing Cereal Research Data.

[105] Vitória A.P., Lea P.J., Azevedo R.A. (2001). Antioxidant enzymes responses to cadmium in radish tissues. Phytochemistry.

[106] Chance B., Maehly A., Colowick S.P., Kaplan N.O. (1957). Methods in Enzymol.

[107] Thomas R.L., Jen J.J., Morr C.V. (1982). Changes in soluble and bound peroxidase-IAA oxidase during tomato fruit development. J. Food Sci..

[108] Fielding J., Hall J. (1978). A biolchemical and cytochemical study of peroxidase activity in roots of Pisum sativum: I. a comparison of DAB-peroxidase and guaiacol-peroxidase with particular emphasis on the properties of cell wall activity. J. Exp. Bot..

[109] Sairam R.K., Rao K.V., Srivastava G. (2002). Differential response of wheat genotypes to long term salinity stress in relation to oxidative stress, antioxidant activity and osmolyte concentration. Plant Sci..

[110] Rao M.V., Paliyath G., Ormrod D.P. (1996). Ultraviolet-B-and ozone-induced biochemical changes in antioxidant enzymes of Arabidopsis thaliana. Plant Physiol..

